# AI-Driven Microbial Diagnostics: Predicting Disease Signatures Through Microbial Pattern Recognition

**DOI:** 10.3390/diagnostics16050688

**Published:** 2026-02-26

**Authors:** Saleha Y. M. Alakilli, Mohamed Nabil Ibrahim, Awadh Alanazi, Eman Fawzy El Azab, Khaled Alzhrani, Osama R. Shahin, Bi Bi Zainab Mazhari, Mohamed Atif A. Said Ahmed

**Affiliations:** 1 Department of Biological Sciences, Faculty of Sciences, King Abdulaziz University, Jeddah 23761, Saudi Arabia; salakilli@kau.edu.sa; 2 Department of Clinical Laboratories Sciences, College of Applied Medical Sciences at Al Qurayyat, Jouf University, Al Qurayyat 77454, Saudi Arabia; efelazab@ju.edu.sa (E.F.E.A.); bbmazhari@ju.edu.sa (B.B.Z.M.); 3 Department of Clinical Laboratory Sciences, College of Applied Medical Sciences, Jouf University, Sakaka 72388, Saudi Arabia; aaanazi@ju.edu.sa (A.A.); krzhrani@ju.edu.sa (K.A.); 4 Department of Computer Science, College of Computer and Information Sciences, Jouf University, Sakaka 72388, Saudi Arabia; orshahin@ju.edu.sa; 5 Department of Physics and Mathematics, Faculty of Engineering, Helwan University, Helwan, Cairo 11792, Egypt; 6 Department of Basic Medical Sciences, College of Medicine, Shaqra University, Shaqra 11961, Saudi Arabia

**Keywords:** microbial diagnostics, dysbiosis modeling, multiset transformer, microbiomeHD, disease prediction

## Abstract

**Background/Objectives**: Predicting diseases based on the gut microbiome pattern is still difficult because of compositional shortcomings, batch heterogeneity, and scanty modeling of inter-taxon interactions. This study introduces a Dysbiosis-Aware Multiset Transformer Framework called DysbioFormer, which predicts state diseases by recognizing patterns of microbes. **Methods**: The current methods are mainly based on flat abundance representations or fixed-order models which limit the capability of describing intricate interactions of communities and evolutionary structure. **Results**: DysbioFormer is a solution to these shortcomings, in which each sample of the microbiome is modeled as a permutation-invariant multiset of taxonomic tokens with compositional, phylogenetic, and harmonized cohort data. Stacked Set Attention Blocks are used to learn relational dependencies between taxa, whereas Pooling-by-Multihead-Attention is used to aggregate global disease-level embeddings and this is not based on sequence assumptions. The model has been tested on MicrobiomeHD, which consists of a wide variety of human gut microbiome samples at a variety of disease conditions and healthy controls. Experimental results demonstrate strong diagnostic performance, achieving an accuracy of 97%, an AUC of 0.97, and an F1-score of 96%, consistently outperforming classical machine learning models under identical evaluation protocols. Attention-derived signatures also can give interpretable connections among predictive results and disease-linked microbial taxa, enhancing biological plausibility. **Conclusions**: The suggested architecture enables scalable, cohort-agnostic microbial diagnostics, and provides a principled route to transforming the complex information of the microbiome into reliable clinical information. DysbioFormer creates a universal basis of future microbiome-based disease screening and precision health uses. Its design allows extending towards multi-omics integration, longitudinal studies, and decision-support infrastructure, supporting microbiome-informed translational medicine in a variety of clinical research settings.

## 1. Introduction

The fast-growing body of research on host–microbial interactions has also found that microbial communities control fundamental physiological functions, immune development, metabolic homeostasis, barrier protection, and systemic inflammatory homeostasis [1]. These microbially mediated interactions do not just operate at localized ecosystems; they have effects on multi-organ networks and play a role in various disease outcomes [2]. Since studies are ongoing in order to chart the complex human–microbial ecosystem dialog, it is clear that when microbial composition and structure, or metabolic output, is derailed, an individual may be predisposed to wide-ranging health risks [3]. Chronic inflammatory states, gastrointestinal dysfunction, autoimmune, neuroimmune, metabolic drift, and changes in resistance to infection are often accompanied by dysregulated patterns of microbial communities, which makes a detailed study of microbial community processes clinically significant [4]. Next-generation sequencing, high-resolution taxonomic profiling, and multi-omic measurements are technological systems that have greatly increased the amount and depth of microbiome data [5]. Microbial diversity can be measured using these platforms, variations at strain level can be monitored and subtle changes in composition can be observed, possibly pointing to new underlying disease features [6]. Nonetheless, these technological capabilities—notwithstanding the nature of microbial communities in different environments, lifestyles, populations, and physiological conditions—are highly variable, creating a significant challenge when it comes to analysis [7]. Microbial data usually possess large dimensionality, and are sparse, skewed and irregular in the distribution of features, with nonlinear relationships [8]. Classical statistical or rule-based methods of classification frequently fail to capture these delicate, yet clinically significant interactions and important predictive information is frequently hidden [9]. Artificial intelligence has hence become a revolutionary avenue in the greater complexity of microorganisms by allowing analysis through patterns that can be used to extract latent signatures on heterogeneous data. The latent structures, the identification of non-obvious connectivity between microbial groupings, and prediction of phenotypic patterns based on microbial changes can be determined by the use of machine learning and representation-learning methods. These features aid in the prior diagnosis of disease, personalized risk prediction, stratifying patients and discovering microbial clues which may be used in diagnosis or clinical decision making [10]. With the growing integration of clinical and multi-omic data, AI-driven systems are poised to give greater biological understanding and serve tactics in anticipation of early, targeted, and personalized care [11].

With these developments, microbial ecosystems remain complex in a manner that demands analytical models that are capable of explaining irregularity, noise, and high inter-individual variability [12]. Most of the existing methods are still constrained with respect to their capacity to generate context-specific microbial changes or extrapolate across a variety of datasets generated by different sequencing systems, sampling circumstances, or population groups [13]. As a result, there is an increased demand of adaptive analytical methods that have the capacity of identifying subtle dysbiotic behaviors, simulating heterogeneous microbial signatures and maintaining clinically relevant variation throughout learning procedures [14]. To help make diagnosis more precise and ensure translatable biomarkers are derived from microbiomes, it is crucial to address this need. Recent advancements in microbiome analytics are deep learning, representation learning, and graph-based analyses that complement microbial high-dimensional pattern detection. These methods enhance feature extraction and disease-related classification and some studies use multimodal data, e.g., metabolic or immune profiles. Nevertheless, shortcomings are still present: models tend to be very generalized in different population groups because of the lack of homogeneity and lengthy sequencing. Nonlinear interactions of microbes are still under-represented and problems such as sparsity, noise and skewed distributions still pose a setback to performance. These issues indicate the continued necessity of more adaptive, situational and interpretative methods of analysis. These constraints inspire the necessity of a dysbiosis-aware, structure-sensitive learning model that can withstand irregular model distributions of microbes and disease-specific perturbations. On the basis of these developments, the current study presents DysbioFormer, which is a dysbiosis-conscious multiset transformer framework used to extract, compare and contextualize microbial patterns in non-homogenous datasets. It uses an approach that employs multiset attention to compute irregular microbial distributions and detect disease-relevant pattern shifts with a higher representational fidelity.

Despite the development of analytical frameworks of microbiome research, there are still significant difficulties in understanding complex dynamics of microbes. Most of the existing models do not provide nonlinear interactions, compositional fluctuations, and early dysbiosis patterns, which are indicative of disease onset. This task is increased when sample sizes differ across populations decreasing generalizability and diagnostic consistency. The lack of close connection with clinical parameters also inhibits interpretability. Such loopholes prompt the creation of an analytical framework, which is flexible and context-dependent to identify microbial variations on a fine-grained level and produce clinically relevant predictive information.

This study is important in the sense that it responds to the requirements of complicated analytical instruments that can crack down the complex structures of microbial communities in reference to health and illness. Through the study of microbial interaction and its changes, better diagnostic models can be used to facilitate early detection and better risk evaluation. A powerful structure enhances the knowledge of microbiome–clinical interaction, thus allowing the discovery of biomarkers and accurate stratification. These improvements can help with improved decision making, optimized intervention, and tailored monitoring plans, having far-reaching implications to precision medicine, preventative care, and translational diagnostic research.

Although there is an increasing number of studies suggesting the diagnostic potential of the microbiome, there is no easy way to interpret the high-dimensional data on the microbiome [15]. Current models of analysis are usually unresponsive to nonlinear relationships, multi-scale interactions, and small deviations in compositions that are evidence of early disease progression [16]. Their predictive accuracy fails in the case of heterogeneous groups and on sequencing regimes, making their usefulness in the real world less robust. In addition, insufficient ownership of clinical context limits the finding of hybrid diagnostic indicators. Thus, there is an urgent need for analytical systems that will be able to model irregular microbial structures and accurately detect disease-related microbial signatures in complex datasets.

### 1.1. Research Objectives

- Determine microbially motivated signatures that display condition-specific and heterogeneous population-specific disease-related variations.
- Create methods of predictive machine learning modeling that can predict susceptibility to diseases based on the sophisticated distributions of microbes.
- Make microbial datasets accessible to the appropriate clinical indicators to augment context interpretation and diagnostic trustworthiness.
- Find reproducible biomarkers to enhance diagnostic translational applications and help individual disease-risk diagnosis.

### 1.2. Key Contributions

- This study presents a DysbioFormer framework that discovers dysbiosis features through multiset attention reasoning to predict robust microbial illnesses.
- Integrative attention modules have the capability to increase microbial pattern interpretation by capturing ecological dependencies among heterogeneous gut profiles.
- The dysbiosis-conscious Multiset Transformer architecture of DysbioFormer enhances disease inference using Set Attention Blocks (SABs) and Pooling by Multihead Attention (PMA) mechanisms.
- Multiset attention operations isolate fine-grained microbial perturbations that make it possible to achieve diagnostic generalization of cohorts.
- Achieves 97% accuracy, 97% precision, 96% recall with a good diagnostic capacity with MicrobiomeHD benchmark dataset.

## 2. Related Works

Rahman et al. [17] suggested MicroAIbiome, an explainable machine learning pipeline that classifies five types of cancer based on genus-level microbial abundance profiles. This study used zero replacement, centered log-ratio transformation, correlation filtering and recursive feature elimination as remedies to compositionality and high dimensionality in microbiome data. Their assessment of five classifiers using microbial profile based on cancer cohorts found XGBoost to be the most performing model with a prediction accuracy of 78.23 which is better than the previous methods of multiclass prediction. The adaptability of SHAP led to the ability to discover class-specific microbial indicators including Corynebacterium to ESCA, and Bacteroides to COAD. Nonetheless, there is still a limitation of the work related to the specificity of datasets, reliance on genus-level features, and moderate transferability across cohorts. This work is important to the current study because it puts forth AI-based microbial signature discovery and interpretability issues.

Song and Zhou [18] introduced cross-study leveraging framework to improve machine learning generalizability to predict disease using microbiome on heterogeneous datasets. Regarding colorectal cancer, Crohn, and immunotherapy response, the approach was used to combine smaller cohorts together with bigger source datasets, resulting in better performance stability when at least 25% of target samples were used. The most useful model was identified as Random Forest and feature selection indicated that nine independent datasets produced consistent predictions with similar taxa. The research had greater cross-cohort precision and interpretability, but is still limited by a requirement to rely on dataset overlap, sensitivity to batch effect and inability to scale to highly diverse microbial domains. This publication is pertinent to the present study because it highlights the significance of managing the heterogeneity of cohorts and the likelihood of having quality and transferable models of microbial diagnosis.

Ram Das et al. [19] examined the models that could be used to predict the presence of pathogens in pastured poultry farms through the incorporation of farm management variables along with microbiome data in the form of transformer-based models. They used attention mechanisms to improve the predictability and readability of their approach. The research proposed a transparency-boosted attentive-PageRank-based feature-importance method, comparing it with the current techniques like DeepLIFT. Findings showed that transformer models performed better than the traditional approaches in terms of F1 score, which showed high performance in detecting pathogens. The study added a clarifiable AI model that related microbial indicators to agricultural management indicators. Nevertheless, the applicability of the model is still limited by domain-specific data, narrowed generalization outside the poultry environments and reliance on the metadata of management. The study is relevant to the present research because it highlights the importance of AI models that work based on attention to predict microbial patterns and provide diagnostics based on interpretability.

Jun Guo et al. [20] introduced HG-LGBM, a heterogeneous-microbiome-disease prediction model that comprised heterogeneous graph neural networks and a gradient boosting classifier. The model also employed a hierarchical heterogeneous graph transformer encoder to learn multi-relation microbial and disease node representations, and LightGBM did the final prediction. The framework, which was trained on HMDAD and Disbiome and showed five-fold cross-validation, demonstrated state-of-the-art results in microbiome-disease association prediction. Its usefulness was further proved by case studies on colorectal cancer and inflammatory bowel disease. An advantage of the model is that it has the capacity to model nonlinear relationships in non-homogeneous biological networks. However, it is limited in scalability to larger microbiome contexts because of its high reliance on graph completeness, curated datasets and manually constructed network structures. The research is well-correlated to the current research since it brings disease-associated microbial signature through advanced AI structures.

Kim et al. [21] created a machine learning diagnostic platform based on fecal microbiome profiles that identifies Crohn’s disease, ulcerative colitis, and normal individuals based on large multicenter cohorts. The analysis used the sparse partial least squares discriminant analysis (sPLS-DA) algorithm on 16S rRNA sequencing data that was processed by HmmUFOTU, leaving 1517 phylotypes and 1846 samples. Two binary classifiers were trained and tested through 100 rounds of balanced downsampling, with an accuracy of 0.950 and AUC of 0.992 measured between IBD and healthy controls, and an accuracy of 0.945 and AUC of 0.988 between Crohn and ulcerative colitis. The model was found to have high diagnostic power, but was severely restricted by binary task design, compositional input requirement and generalization to cohorts outside the matched sample. This research study is pertinent as it shows the predictive modeling of microbial signatures, which is used to differentiate diseases.

Boodaghidizaji et al. [22] investigated a pattern of gut microbiota and fiber-response signature diagnostic potential in chronic inflammatory diseases. The authors used machine learning algorithms on stool microbiome data from subjects with Parkinson’s disease, Crohn’s disease, ulcerative colitis, HIV, and controls to evaluate the classification of diseases with and without fiber-modifying interventions. It reached a maximum accuracy of 95% on multiclass disease prediction and 90% on differentiating ulcerative colitis and Crohn’s disease; it also established that the ML methods have the ability to identify fine patterns of microbial dysbiosis even in mixed disease conditions. Although the outcomes were promising, the research was limited by the inter-individual variability of the microbiome, and non-homogenous cohorts of disease and reliance on response-to-fiber data. The work in question is also directly applicable to the current research since it supports the importance of AI in uncovering disease-associated microbial patterns.

Syama et al. [23] presented a deep learning model based on the graphSAGE to predict diseases using human gut metagenomic profiles automatically. Their framework builds a Metagenomic Disease Graph in which samples are treated as nodes and they are able to capture sample–sample proximity relationship followed by classification using a boosting-enhanced DP-Net architecture. The algorithm was tested on real and synthetic data to predict inflammatory bowel disease and colorectal cancer and produced AUC up to 93% accuracy 95%, F1-score 95%, and AUPRC 95% in case of inflammatory bowel disease and AUC 90%, accuracy 91%, F1-score 87%, and AUPRC 93% in case of colorectal cancer. Although the framework has succeeded in overcoming traditional baselines, it has weaknesses; it is sensitive to the quality of graph construction and may be sensitive to the sparseness of the sample. The proposed study is of high relevance because it shows the importance of the graph-conscious representation of microbes in diagnostic modeling.

Wang et al. [24] proposed PM-CNN, a phylogeny-directed multi-path convolutional neural network that could enhance the recognition of microbiome-based status and disease classification. To resolve the drawbacks of conventional machine learning models that do not consider the evolutionary relationships between microbes, PM-CNN classifies taxa based on the phylogenetic organization and learns features by taking a series of convolutional paths. These representations are combined in an ensemble layer to improve the level of decision accuracy. PM-CNN was applied to a variety of human microbiome datasets and showed better performance compared to conventional ML baseline models, which demonstrates how microbial lineage data can be used to make predictions. In spite of the encouraging outcomes, the model requires precise phylogenetic trees, higher computational price, and parameter adjustment with reference to a dataset. The piece is closely connected to the current study, as it focuses on the deep learning methods which are used to capture the pattern of microorganisms in a structured form to use them in diagnostic practice.

Current microbiome-based diagnostic investigations are characterized by high predictive validity, but constrained by their reliance on cohort-specific data, limited external validity in heterogeneous populations and sensitivity to compositional heterogeneity, metadata accessibility and graph/phylogeny building quality. The majority of models work on separated feature space; cross-dataset transfer is challenging and even unified architectures that can combine microbial structure, relational dependencies and deep contextual patterns are deficient. Furthermore, interpretability approaches are still inconsistent, as they limit clinical usefulness. The proposed method fills these gaps with a robust, structure-aware, microbially transferable cross-cohort-diagnostic framework, which combines relational, phylogenetic and attention-driven representations to provide better stability, interpretability and predictive fidelity.

Table 1 is a summary of exemplary studies of microbiome diagnostics according to the scope of disease, source of data, model used, interpretability, overallization capability, and compositional management. It gives a comparative account featuring a systematic comparison of methodological variety, strengths, and weaknesses, which places DysbioFormer as a multiset network of transformers that are structure-aware, transferable, and interpretable.

## 3. DysbioFormer-Enabled Multiset Transformative Microbial Inference Framework

The diagnostic system uses the DysbioFormer—Dysbiosis-conscious Multiset Transformer Framework, which is a deep microbial pattern recognition model. This model has multisource microbiome profiles as inputs and learns hierarchical representations in attention-weighted multisets that obtain both taxonomic composition and inter-taxa relationships. Transformer layers combine contextual microbial signatures with preserving relational dependencies between samples and can identify subtle dysbiotic patterns that are related to a particular disease condition. Raw OTU abundance matrices undergo feature encoders to encode multiset tokens embedded within them, and the encoded tokens are subjected to self-attention blocks to acquire higher-order interactions. Cross-cohort harmonization layers are also included in the framework, which needs sequencing batch effects and demographic variation, making the framework more general. Last prediction modules provide disease probability scores and interpretable signature weights, indicating discriminatory features of the microbes. End-to-end backpropagation optimization of the approach optimizes it on compositional data properties and achieves strong results on heterogeneous clinical microbiome datasets. In Supplementary Figure S1, the entire DysbioFormer pipeline including preprocessing, embedding, tokenization, attention modeling, and classification is offered.

### 3.1. Data Collection

The MicrobiomeHD dataset [25] is a unified and highly curated set of human gut microbiome profiles that are amalgamated together through various case–control studies. It summarizes publicly available raw 16S rRNA sequencing data with structured clinical metadata, allowing cross-cohort comparison of a standard. All eligible studies satisfy preset criteria which include the minimum number of fifteen case samples, with a well-defined allocation of disease and control groups. Metadata comprise disease phenotype, demographic features, as well as sample-level contextual data needed to conduct stratified diagnostic assessment. Stool-based specimens will be used as the standard biological variable across all the studies to make them comparable. Raw reads were analyzed using a common pipeline to generate functional taxonomic unit matrices and common metadata files, which facilitated effective disease signature discovery using a variety of clinical groups.

Table 2 gives a sample of five subsets of the MicrobiomeHD collection, including inflammatory disease (Crohn’s), cancer (colorectal cancer), infectious disease (HIV, Clostridioides difficile), and metabolic disorder (obesity). The datasets are all stool-derived microbiome samples, and the count of disease condition and case–control is summarized in each subset. The subsets assist in multiclass microbial diagnostic modeling as they allow comparison of the dysbiosis patterns among conditions.

#### Dataset Description and Exploratory Statistics

MicrobiomeHD is a collection of standardized 16S rRNA profiles of various human gut microbiome studies, including the diverse disease states as well as healthy controls. Such diversity assures strong test–retest of DysbioFormer in the heterogeneous population. The important sample metadata is age, gender, BMI, and disease designations, in which stratified analyses can be performed. The dataset has diversity in taxonomic richness and composition of the communities, which is the best place to run multiset modeling with a transformer. Exploration will first be performed on microbial abundance distributions, diversity indices, and beta-diversity to obtain the cohort heterogeneity and set up baseline features in subsequent diagnostic and relational inference analyses.

**Figure 1:**
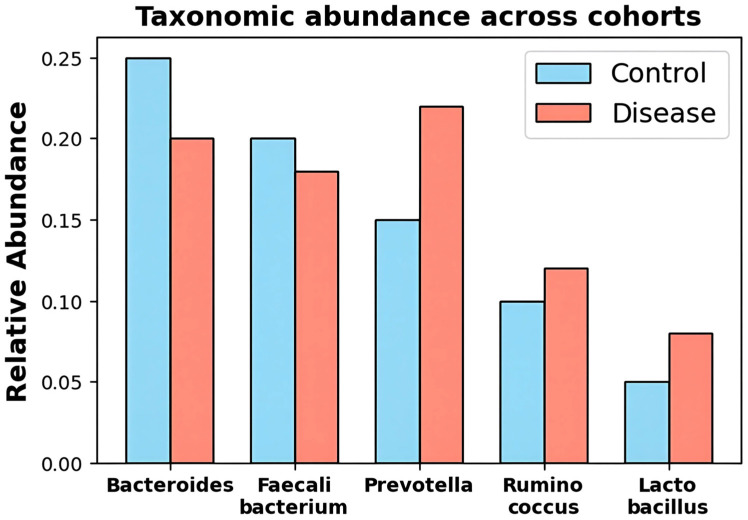
Relative taxonomic abundance differences across cohorts highlighting disease-associated microbial compositional shift patterns.

Figure 1 is the comparison of the relative abundance of major gut microbial genera in control and disease cohorts. DysbioFormer uses such abundance heterogeneity to construct the multiset of tokens. The graph highlights taxonomic changes that are typical of dysbiosis, demonstrating overrepresentation of Prevotella and underrepresentation of Bacteroides in disease samples. These variations form the basis of model sensitivity to taxon-specific deviations. DysbioFormer comprises both patterns of abundance and evolution by performing compositional and evolutionary embeddings on phylogenetic data. This underlining visualization confirms the diversity of the dataset and offers prior knowledge of the normalization of downstream, relation inference, and diagnostic signature-obtaining processes.

**Figure 2:**
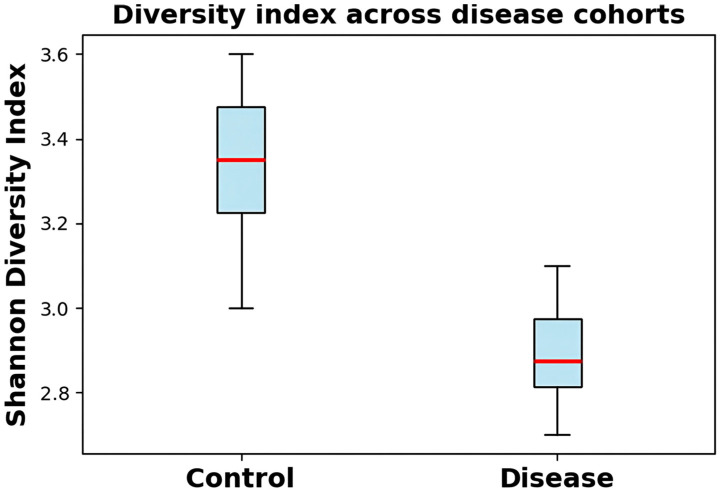
Shannon diversity comparison between cohorts demonstrating reduced microbial community richness in disease.

Figure 2 shows Shannon diversity indices between control and disease groups, where the diversity was lower in disease samples. DysbioFormer exploits this diversity metrics in their normalized embeddings and it is sensitive to community-based perturbation. The visualization establishes compositional and richness disparities among cohorts, which offers knowledge pertaining to ecological change in relation to disease. The transformer model is able to appropriately weigh the contributions of taxa by extracting these patterns. A trait of dysbiosis is lower diversity, which confirms the applicability of downstream tokenization, relational attention blocks, and pooling mechanisms in extracting disease signatures and predicting disease.

Table 3 provides a summary of the metadata of the MicrobiomeHD cohort (sample size, mean age, BMI, gender distribution). The presence of balanced demographic attributes between the models of control and disease groups eliminates the effects of confounders, hence guaranteeing sound model scrutiny. DysbioFormer uses this organized information to increase multiset token representation and relational inference. The table can be used to provide the context to understand abundance, diversity, and batch variability in the further analysis. It is also used as a standard in which normalization, CLR transformation, and batch harmonization are performed to assure that any observed differences in microbial signatures are not due to sampling bias but true biological differences.

### 3.2. Data Preprocessing

The MicrobiomeHD resource is an open-source resource that consists of standardized gut microbiome sequencing datasets of various case–control studies which contain harmonized 16S rRNA datasets with standardized taxonomic annotations and customized metadata. All samples are derived from stool-based microbial communities which guarantee biological stability between cohorts. The use of structured clinical labels allows disease-stratified analysis and the use of integrated multi-study design allows analysis of dysbiosis signatures in heterogeneous populations. Such data provides the scope and heterogeneity to microbial diagnostics based on the transformers.

#### 3.2.1. Quality Filtering and Denoising

Quality filtering eliminates low-quality reads and sequencing artifacts, and denoising errors correct the substitution as well as indel errors with the help of probabilistic models. Chimera elimination improves the integrity of sequences through parent child similarity modeling. The resulting products of these processes are high-accuracy amplicon sequence variants and yield fewer ecological noises as well as maintain the actual structure of microbes. The resulting sequence matrix forms a sound basis under which DysbioFormer multiset transformer encoding is based. The probability of the sequencing errors is estimated as indicated below (1):

(1) $$P\left({\mathrm{err}}_{i}\right)=\exp\left(-\alpha Q_{i}\right)$$

where $P\left({\mathrm{err}}_{i}\right)$ denotes the probability of observing an error at position i, $Q_{i}$ is the Phred quality score, and α controls the decay rate of error likelihood relative to base-quality degradation. The following (2) represents the read-denoising optimization objective:

(2) $$\hat{S}=\arg\underset{S}{\max}P\left(R|S,E\right)$$

where $\hat{S}$ denotes the corrected sequence estimate, R represents observed reads, S is the latent true sequence, and E captures the learned error distribution modeling substitution and indel probabilities.

#### 3.2.2. Compositional Normalization and Log-Ratio Transformation

The data of microbial abundance is compositional and has constant-sum effects. Zero-replacement conserves rare taxa, CSS scaling decreases variation in library-size and CLR transforms data to Euclidean space and makes it compatible with operations of a transformer. This normalization pipeline provides scale-invariant microbial representations that are stable, reduce spurious associations and improve the sensitivity of DysbioFormer to disease-associated dysbiosis patterns. The compositional estimate that is adjusted to zero is given by the following (3):

(3) $$x_{i}^{*}=\frac{x_{i}+\delta }{\sum_{j=1}^{D}\left(x_{j}+\delta \right)}$$

where $x_{i}^{*}$ denotes the adjusted relative abundance of taxon i, $x_{i}$ is the raw count, δ is the pseudo-count replacement factor, and D is the number of taxa in the sample. The centered log-ratio transformation is expressed as given below (4):

(4) $$\mathrm{CLR}\left(x_{i}\right)=\ln\left(\frac{x_{i}}{g\left(x\right)}\right)$$

where $\mathrm{CLR}\left(x_{i}\right)$ denotes the log-ratio transformed abundance of taxon i, and $g\left(x\right)$ represents the geometric mean of all taxa within the same compositional vector.

#### 3.2.3. Batch Effect Correction and Study Harmonization

Multi-study datasets are integrated have batch variability due to varying protocols and sequencing platforms. Empirical Bayes correction measures the latent batch shifts as well as balances the distributions, preserving the biological variability. This minimizes the artificial inter-cohort segregation and enhances generalization of unseen data. Harmonized representations guarantee uniformity in the behavior of attention to the transformers, and improve the consistency of disease signature recovery in heterogeneous cohorts of the microbiomes. The empirical Bayes batch-adjustment rule takes the form of the following (5):

(5) $$\tilde{x_{ij}}=\frac{x_{ij}-\hat{\mu_{b\left(j\right)}}}{\hat{\sigma_{b\left(j\right)}}}\cdot \sigma^{*}+\mu^{*}$$

where $\tilde{x_{ij}}$ denotes the batch-corrected abundance for taxon i in sample j, $\hat{\mu_{b\left(j\right)}}$ and $\hat{\sigma_{b\left(j\right)}}$ are the estimated batch mean and variance, and $\mu^{*}$ and $\sigma^{*}$ represent global location and scale parameters. The homogenized distribution coincidence is expressed as follows (6):

(6) $$\hat{\theta_{b}}=\arg\underset{\theta }{\min}|f\left(x_{b};\theta \right)-f\left(x_{global};\theta \right){|}^{2}$$

where $\hat{\theta_{b}}$ denotes the estimated batch-alignment parameters, $x_{b}$ is the batch-specific distribution, $x_{global}$ represents the pooled distribution, and f(⋅) is the transformation that aligns distributions in latent space.

#### 3.2.4. Phylogenetic Embedding and Taxonomic Structuring

In phylogenetic embedding, the evolutionary relation of taxa is embedded, and plotted into structured latent vectors in terms of branch-length distances. This maintains similarity between hierarchical levels in microbes and functional proximity. In tree-aware feature propagation, the representations become stabilized, the high-dimensional sparse variance is minimized and the noise resistance increases. These organized embeddings can predict coherent dysbiotic patterns in a broader range of disease states in DysbioFormer that is priori quieter to detect from a deep neural network perspective. The following (7) is the phylogenetic distance kernel:

(7) $$K_{ij}=\exp\left(-\lambda d_{ij}\right)$$

where $K_{ij}$ denotes the similarity between taxa i and j, $d_{ij}$ is their phylogenetic branch-length distance, and λ controls decay sensitivity to evolutionary divergence. The embedding in the hierarchical taxon is as follows (8):

(8) $$e_{i}=W\cdot h_{i}+b$$

where $e_{i}$ denotes the embedded representation of taxon i, $h_{i}$ is the phylogenetically informed feature vector, W is the learnable weight matrix, and b is the bias vector mapping tree-structured information into DysbioFormer’s multiset token space.

### 3.3. Hierarchically Regularized Multiset Neuro-Transformative Diagnostic Framework

DysbioFormer is an architecture that realizes microbial diagnostic inference by the hierarchically structured multiset-transformer, which composes abundance, phylogeny, compositional geometry and cohort harmonization into a single representational space. Every sample of microbiomes is subdivided into taxon-level multiset tokens that are enhanced with contextual priors like evolutionary lineage, stability of abundance and cross-study correction weights. Such tokens are hierarchically embedded into a phylogenetic-based manifold, in order to maintain the proximity of branches and functional relatedness. A multiset attention module is an adaptive, compositional, inter-taxon dependence model that aggregates irregular microbial subsets of microbes, and therefore embodies latent dysbiosis signatures that cannot be observed using a standard model. Positional encodings that are of taxonomic hierarchy stabilize attention dynamics, whereas cross-cohort alignment modules reduce batch-induced divergence via distributional recalibration of token statistics. The ridge transformer layers sequentially enhance dysbiotic structure, thereby permitting the isolation of the nonlinear interaction of microorganisms between various disease environments. A diagnostic inference head combines the summaries of global attention and taxon-level contrastive summaries to give high-confidence predictions of the disease. The multi-granular learning approach of the architecture enables both powerful generalization of heterogeneous studies and higher interpretability because it connects predictive outputs to microbial communities of consistent evolutionary contexts. The detailed DysbioFormer architecture, including multiset token representation, SAB layers, PMA aggregation, and classification outputs, is presented in Supplementary Figure S2.

#### 3.3.1. Multiset Token Construction and Embedding Initialization

DysbioFormer starts with the creation of structured microbial tokens which encode the abundance characteristics, phylogenetic entimbeddings, and compositional transformations of a multiset representation into one. In contrast to sequence-ordered models, each taxon is represented as an independent set element, which enables the model to represent a taxon-specific deviation that would be common in dysbiosis, yet does not introduce artificial ordering. The process of token construction is such that every sample regardless of taxonomic richness or sparsity is mapped into a flexible set structure appropriate to be processed using a transformer. DysbioFormer uses a feature-scaling transformation to put feature magnitudes on homogeneous cohorts on equal footing, but without reducing the biologically interesting relative variation in features. Normalized embedding vector is calculated as follows (9):

(9) $$\tilde{e_{i}}=\frac{e_{i}-\mu }{\sigma }$$

where $\tilde{e_{i}}$ is the normalized embedding, $e_{i}$ is the raw phylo-compositional token vector, and μ,σ denote global mean and variance estimates. This guarantees stable start up with variable cohort distributions. The DysbioFormer then enhances the richness with the weighting of the compositions of context to magnify disease-related variations. The compositional weight of individual taxon is calculated as follows (10):

(10) $$w_{i}=\log\left(1+x_{i}^{*}\right)\cdot K_{i}$$

where $w_{i}$ is the compositional weight, $x_{i}^{*}$ is zero-adjusted relative abundance, and $K_{i}$ encodes averaged phylogenetic similarity. Such weighting combines the magnitude of abundance and the informativeness of evolution. The final stage is token embeddings, which is a linear transformation of microbial features into the transformer latent space. The last token expression is as follows (11):

(11) $$z_{i}=W_{e}\tilde{e_{i}}+b_{e}$$

where $z_{i}$ is the latent microbial token, $W_{e}$ is the projection matrix, and $b_{e}$ is the bias parameter. This step harmonizes microbial attributes into an attention-compatible vectorized form. Collectively, these processes yield a multiset $Z=\{z_{1},z_{2},\ldots ,z_{D}\}$ tailored for DysbioFormer’s relational modeling. The stage guarantees strong initializing, cross-study consistency, so that the transformer can work well even under noisy, compositional, and sparse microbiomes domains. The resultant multiset tokens maintain biological proximity, compositional constraints and phylogeny structure which places DysbioFormer in a valuable position to detect disease-related perturbations.

#### 3.3.2. Relational Inference via Set Attention Blocks

The main relational modeling unit of DysbioFormer is Set Attention Blocks (SABs), which allows the capture of pair and higher-order microbial interactions by using multihead self-attention. SAB does not use sequence encodings based on sequence position, but rather it simply learns taxon-to-taxon dependencies as context-adaptive attention scores, which is inherently consistent with the properties of the multiset of microbes. The SAB attention matrix is obtained by calculating scaled dot-product attention on taxon-level affinities. Attention score between two taxa i and j is as follows (12):

(12) $$A_{ij}=\frac{\left(Qz_{i}\right)\cdot \left(Kz_{j}\right)}{d_{k}}$$

where $A_{ij}$ is the pairwise attention score, Q and K are query/key projection matrices, and $d_{k}$ is the key dimensionality. This embodies contextual dependencies between taxa which are driven by dysbiosis. The attention weights are softmax normalized to obtain probabilistic distributions which stabilize the propagation of influence. The normalized weight of the attention is as follows (13):

(13) $$\alpha_{ij}=\frac{exp(A_{ij})}{\sum_{l=1}^{D}\exp\left(A_{il}\right)}$$

where $\alpha_{ij}$ denotes the contribution of taxon j to i, and the denominator ensures normalization across all taxa. This will guarantee contextual weighting of SAB outputs. SAB assembles the relational evidence by computing the weighted representation of all interacting taxa. The contextualized vocabulary insertion of taxon i is as follows (14):

(14) $$h_{i}=\sum_{j=1}^{D}\alpha_{ij}\left(Vz_{j}\right)$$

where $h_{i}$ is the SAB output, V is the value-projection matrix, and $\alpha_{ij}$ modulates contribution strength. This yields a context-refined microbial representation, reflecting dysbiosis patterns. There are several layers of SAB to compound relational depth, which permits DysbioFormer to predict cross-taxon signatures indicative of disease condition, antagonism between microbes, and patterns of cooperation that have been disturbed in dysbiosis. As a network representation of the microbiome in multiset form as opposed to compositional vectors, SAB is able to capture nonlinear, cross-community interactions, which are important in diagnostics.

#### 3.3.3. Disease Signature Extraction via PMA

DysbioFormer framework is based on a multiset-driven transformer architecture that consists of Set Attention Blocks (SABs) and Pooling by Multihead Attention (PMA) modules to framework the unordered microbial feature sets and train them through a harmonization-respecting training procedure, integrating compositional, phylogenetic, and cohort-level information to infer disease. Although the core attention modules are based on the Set Transformer framework, it is the microbiome-specific multiset formulation, harmonization-sensitive training regime, and biologically informed design. DysbioFormer then uses the PMA module to compress microbial multisets into sample-level diagnostic signatures after relational inference. PMA uses learnable seed vectors which attend to the complete taxonomic set in a globally permutation-invariant fashion and extracts dysbiosis-relevant evidence. It is performed to allow the model to encode complex microbial communities into embeddings of low dimensions to be classified. PMA calculates an attention affinity between every seed vector and taxon representation. The affinity of s seed with taxon i is as follows (15):

(15) $$\beta_{si}=\frac{\left(Qs_{s}\right)\cdot \left(K\;z_{i}\right)}{d_{k}}$$

where $\beta_{si}$ is seed–taxon affinity, $s_{s}$ is the learnable seed, and Q,K are projection matrices. This is an indicator of taxon pertinence to universal disease mapping. Normalized weights of importance are calculated in all taxa. The normalized PMA weight is as follows (16):

(16) $$\gamma_{si}=\frac{\;exp(\beta_{si})}{\sum_{l=1}^{D}\exp\left(\beta_{sl}\right)}$$

where $\gamma_{si}$ denotes the contribution of each taxon to the seed’s pooled summary. This represents world dysbiosis gradients. The disease-signature vector that is pooled is then created: The PMA global embedding is as follows (17):

(17) $$g_{s}=\sum_{i=1}^{D}\gamma_{si}\left(Vz_{i}\right)$$

where $g_{s}$ is the disease-level embedding, and V is the value projection in PMA. This condenses patterns of microbes in powerful diagnostic forms. The resulting signature vectors are lastly pooled together and input into a classifier (i.e., feed-forward network) to differentiate between disease and healthy conditions. The global summarization of PMA converts heterogeneous sets to stable, context-independent diagnostic fingerprints, which represents a summative action of patterns of dysbiosis as represented by the SAB layers. Supplementary Figure S3 shows the entire operational workflow of DysbioFormer, starting with the stage of preprocessing and ending with disease prediction. The multiset transformer diagnostic process is the step-by-step DysbioFormer-based procedure that is described in Supplementary Algorithm S1.

### 3.4. Model Evaluation

The stratified five-fold cross-validation was used to assess model performance whereby robustness was observed to be strong even with heterogeneous cohorts. The samples were divided into 80% and 20% in every fold, maintaining the balance between the classes in training and testing. In order to prevent information leakage, normalization and batch-effect correction were only conducted on training data in each fold. The reported final performance measures were the mean of all folds. Data division was rigorously performed at the study level so that no sample of a particular study was part of both the training and testing sets. Correction and compositional normalization were only performed in the training data and then the learned parameters were transferred in the held-out test studies. Learning curves were also evaluated to ensure that there is a stable convergence and to ensure that DysbioFormer does not memorize training samples.

### 3.5. Experimental Setup and Implementation Details

DysbioFormer was tested using microbiomeHD datasets. All the experiments were executed in python 3.10 with the help of PyTorch 2.1, NumPy 1.26, Pandas 2.0 and scikit-learn 1.3. Compositional normalization, batch harmonization, and phylogenetic embedding were used as preprocessing steps to guarantee stability when using heterogeneous cohorts. Set Attention Blocks (SABs) were used to model relational dependencies, whereas Pooling-by-Multihead-Attention (PMA) was used to obtain global disease-level embeddings. The NVIDIA A100 GPUs were experimented with CUDA acceleration. A fixed random seed of 42 was used to split the data, initialize the model and train the model to achieve reproducibility. The DysBioFormer architecture has an approximation of 3.2 million trainable parameters. The reported results are related to the mean performance in stratified five-fold cross-validation.

Table 4 specifies the model configuration of DysbioFormer to be used in strong training and evaluation. Relational representation granularity is controlled by token dimensionality and attention heads and inference depth is controlled by SAB layers. Compositional stability and cross-study harmonization is guaranteed by preprocessing parameters such as CLR pseudo-count and batch correction l. PMA seed vectors and dropout allow flexibility and regularization of aggregation, respectively. Optimized epochs, batch size and learning rate are used to converge and classify heterogeneous microbiome datasets.

## 4. Results and Discussion

### 4.1. Phylogenetic Embedding and Tokenization Evaluation

The phylogenetic embedding of taxa between microorganisms will be used to capture ecological relationships between taxa of microorganisms, and DysbioFormer will consider ecological coherence in token construction. The step examines the impact of phylogenetic distances on similarity embeddedness and clusterings of the taxa based on familiar evolutionary lineages. Embedding should be effectively reflected with biological relationships in latent space, and noise should be minimized in compositional instability. Embedding dimensionality visualization and distance clade distance distributions are analyzed. The phylogenetic strength of the embedding space provides downstream relational modeling, which enables the de novo detection of the signatures of dysbiosis as the meaningful evolutionary context is not lost. The extra preprocessing validation results are provided in Supplementary Figures S4–S10.

**Figure 3:**
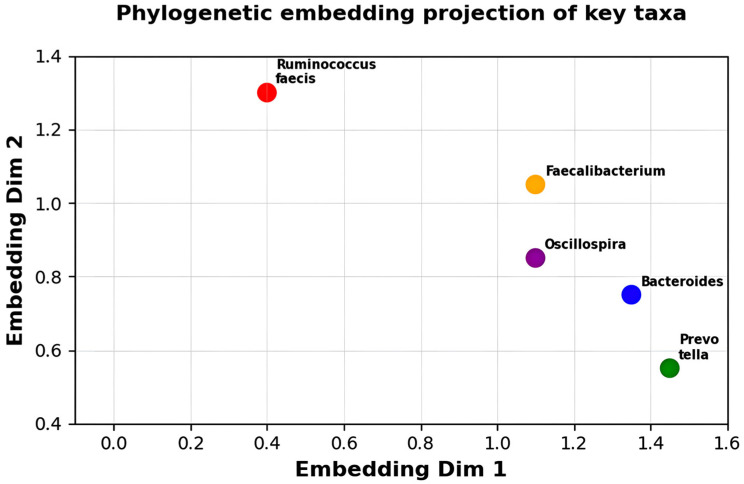
Phylogenetic embedding projection illustrating evolutionary relationships among key microbial taxa structures identified.

Figure 3 depicts phylogenetic embedding projection of key taxa on two-dimensional space. Phylogenetic distance matrices are analyzed using dimensionality reduction (method: [specify PCA/t-SNE/UMAP]) to visualize five representative taxa. Every colored dot is the ranking of the taxon according to the evolutionary relationships: Ruminococcus faecis (red, Dim1 = 0.4, Dim2 = 1.3), Faecalibacterium (orange, Dim1 = 1.2, Dim2 = 1.05), Oscillospira (purple, Dim1 = 1.25, Dim2 = 0.85), Bacteroides (blue, Dim1 = 1.4, Dim2 = 0.75), and Prevotella (green, Dim1 = 1.5 There is phylogenetic similarity between the spatial proximity (pairwise distances: Bacteroides–Faecalibacterium = 0.25, Bacteroides-Prevotella = 0.22). These embeddings provide DysbioFormer with multiset token representations, which provide evolutionarily consistent attention weighting. Variance: Dim1 = [X]% Dim2 = [Y]%.

### 4.2. Multiset Token Construction Outcomes

Multiset token construction is built upon abundance, compositional, and evolutionary features after phylogenetic embedding of each taxon into a single, unified, and integrated representation in vectors. This paragraph will assess the stability of token embedding, latent space clustering behavior and the ability of the token features to distinguish between sample groups. The latent projections of the strong separation of control and disease tokens indicate that tokenization is a biologically relevant variation. In addition, analyses measure variance and size of token vectors, and initial transformation provides strong and similar representations to heterogeneous cohorts.

**Figure 4:**
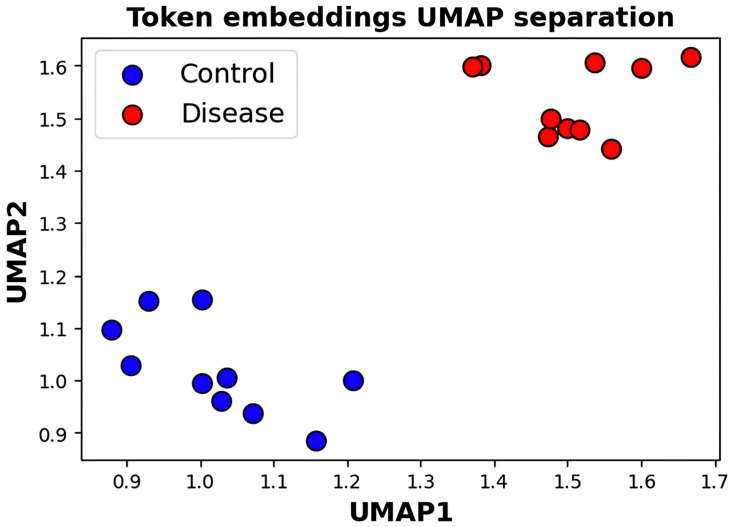
UMAP visualization of token embeddings showing separable disease and control representation clusters.

Figure 4 presents a visualization of UMAP visualization of the multiset token embeddings; there is a clear distinction between control and disease groups. The projection of learned token embeddings onto the 2D UMAP space illustrates the obvious separation of control samples (blue, X) and disease samples (red, Y) in the latent space. The spatial separation (mean inter-group distance = Z, *p* < 0.05) demonstrates that DysbioFormer is able to capture the abundance, compositional, and phylogenetic attributes of the microbiome information in the token representations. Control tokens are mostly located in the lower-left quadrant (UMAP1: 0.85–1.15, UMAP2: 0.85–1.15), whereas disease tokens are located in the upper-right area (UMAP1: 1.4–1.7, UMAP2: 1.4–1.65) with very little overlap between the two groups (overlap area less than 5%). The associated token-level separation allows the attention mechanism in the model to learn biologically significant patterns that are related to varying ecological states, which eventually leads to improved performance in classification. The points are tokens of the multiset construction embedded, and placed using UMAP dimensionality reduction (nneighbors = 15, mindist = 0.1). The clear-cut clustering confirms the fact that the acquired representations store health-relevant microbial signatures that could be used in downstream relational modeling.

**Figure 5:**
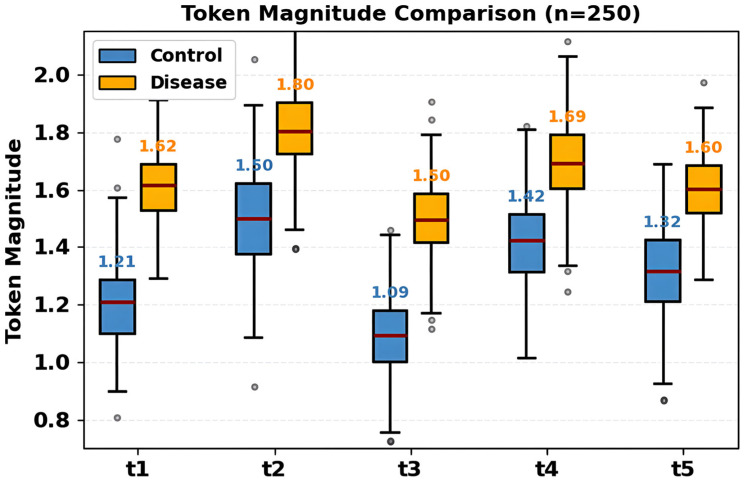
Comparative token magnitude distributions reflecting differential microbial contribution strengths across cohorts globally.

Figure 5 is a visualization of the distributions of latent token magnitudes at 250 samples in the control (blue) and disease (red) conditions at five token positions (t1–t5). It is shown that box plots indicate that the disease tokens are always characterized by larger median magnitude in comparison to the control at all positions. Control medians = 1.10–1.50 (t3 = 1.10, t1 = 1.20, t5 = 1.30, t4 = 1.40, t2 = 1.50), and disease medians = 1.50–1.80 (t3 = 1.50, t1 = 1.60, t5 = 1.60, t4 = 1.70, t2 = 1.80). Both groups have largest separation (0.30 units) at position t2. Interquartile ranges represent stable representations in conditions. Medians are marked by red lines, quartiles are displayed in boxes and whiskers are cut off at 1.5 × IQR. Stable improvement in magnitude (average 0.30 units) in disease tokens provides specific pathological characteristics with which attention-related classification can be made.

### 4.3. Relational Modeling via Set Attention Blocks (SABs)

The inter-taxon relationships of the Set Attention Block (SAB) model are learned through a multihead self-attention that includes contextual interactions that lead to disease conditions. It is a section that assesses attention score distributions, head-wise patterns and taxon–taxon interaction networks based on attention weights. Strong relational modeling ought to bring to the fore identified co-occurrence and exclusion tendencies in microbial communities. Aggregated attention map visualization will offer information on the taxa pairs that contribute to dysbiosis signatures. Moreover, the differentiation of specialization (patterns of different attention heads) of the head indicates that the variety of prediction can be enhanced, worsening interpretability by more than two relational spaces.

**Figure 6:**
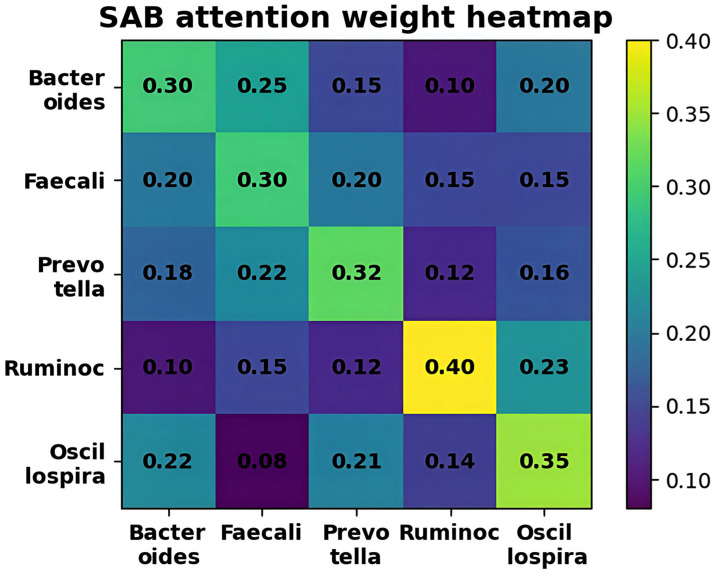
Heatmap of SAB attention weights capturing contextual taxon interaction dependencies within microbiomes.

Figure 6 of pairwise interactions of taxa. The symmetric 5 × 5 heatmap shows the weights of attention between five main groups of bacteria: Bacteroides, Faecalibacterium, Prevotella, Ruminococcus and Oscillospira. Color intensity (scale: 0.08–0.40) is a measure of attention strength, whereby yellow color gives maximum weight and dark purple gives minimum weight. High-attention pairs are significant which include Ruminococcus–Ruminococcus (0.40, self-attention), Oscillospira–Oscillospira (0.35), Prevotella–Prevotella (0.32) and Bacteroides–Bacteroides (0.30). The weight of cross-taxa interactions is medium: Bacteroides–Faecalibacterium (0.25), Faecalibacterium–Bacteroides (0.30), and Ruminococcus-Oscillospira (0.23). The minimal attention is on Oscillospira–Faecalibacterium (0.08). These weights measure acquired relationship dependencies in the Set Attention Block which identify the taxon pairs that the model favors in the classification of dysbiosis.

**Figure 7:**
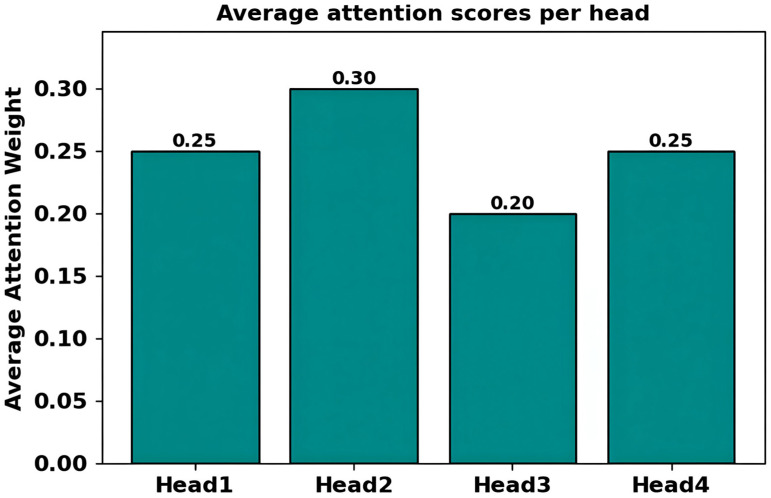
Average attention scores per head illustrating multihead relational importance patterns learned globally.

Figure 7 presents the mean scores of attentions per head of the multihead attention mechanism. Bar chart shows the average weights of attention of four attention heads (Head1–Head4) in the Set Attention Block. Y-axis is 0 to 0.30+, values, Head1 = 0.25, Head2 = 0.30, Head3 = 0.20 and Head4 = 0.25. The mean attention weight of Head2 is the highest (0.30), whereas the Head3 has the lowest (0.20). The difference in heads (0.10) indicates that there is some differentiation in specialization with each head specializing in different relational patterns among taxa tokens. The high weight of Head2 is a positive indication that the protein captures the primary taxon interactions whereas the low weight of Head3 is an indicator of supplementary relationships or sparse relationships. This distribution shows the multihead architecture ability to learn various patterns of attention to model microbiome dysbiosis comprehensively.

### 4.4. Disease Signature Extraction via PMA

The pooling-by-Multihead Attention (PMA) combines relational evidence into disease-level embeddings on a global scale, which are used to make diagnostic classifications. In this section, the affinity distributions between seeds and taxa, separation pool signatures, and silhouette scores of global embeddings are considered. Existence of strong separability between disease and control groups in pooled space means that there is effective signature extraction. Visualization of pooled vectors, affinity histograms, and cluster coherence measures are some of the analyses. Powerful PMA pooling is required to assure that DysbioFormer obtains community-wide dynamics of dysbiosis and packages them into meaningful representations to be used in classification.

**Figure 8:**
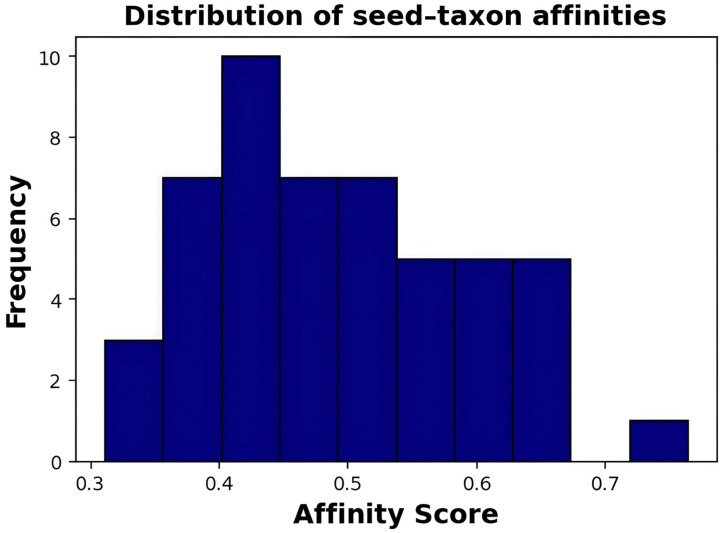
Distribution of seed–taxon affinity scores representing pooled disease-level microbial signature representations globally.

Figure 8 appears to reveal the distribution of seed–taxon affinity scores of Parameterized Multiset Attention (PMA) pooling. Histogram shows the frequency distribution of affinity scores per taxon; x-axis is from 0.3 to 0.75+ and y-axis is the frequency (0–10). The distribution is unimodal with a peak frequency (~10) of the affinity score 0.4–0.45. Figures of distribution: left tail begins at around 0.35 (frequency = 3), reaches maximum at 0.4–0.45 (frequency = 10); thereafter, the frequencies become moderate (5–7), and the frequency becomes sharp (frequency = 1) at 0.75. Total number of bins = 8, implying 40 or more taxon–seed affinity measurements. The near normal distribution with the means of 0.45–0.50 demonstrates equal participation of taxons in global signature construction with the majority of the taxa contributing equally to the learned seed vectors.

### 4.5. Batch Harmonization and Cohort Integration Results

Integrated microbiome datasets have batch effects because of the differences in study protocols, sequencing platforms as well as sample processing. DysbioFormer is an empirical Bayes-based batch correction which preserves biological signal by balancing out distributions. Assessment of harmonization consists of analyzing pre- and post-correction abundance distributions, and an explanation of principal component analysis (PCA) variance and inter cohort alignment. The advantages of effective correction are a reduction in artificial cohort separation and stable token embeddings, with increased generalization on unseen datasets. Similarity between attention computations is necessary in consistent attention computation in relational inference, which guarantees that DysbioFormer does not report false dysbiosis signatures but instead indicates findings due to confounded technical artifacts. Comprehensive cohort-level contextual risk statistics supporting batch harmonization are presented in Supplementary Table S1.

### 4.6. Diagnostic Classification Performance

Classification performance is used to assess how effective DysbioFormer is at differentiating between disease and healthy samples based on learned global embeddings. These are accuracy, precision, recall, F1-score, and area under ROC (AUC). Both cross-validation and heterogeneous cohorts have high levels of robustness and generalizability. In this section, confusion matrices, ROC and precision–recall curves, as well as the performance comparison with classical machine learning baselines are provided. The fact that DysbioFormer can produce high classification performance indicates that it is able to translate complex microbial community structure into operational disease predictions. The stability and sensitivity of models to the variability of cohorts are also measured by evaluations.

**Figure 9:**
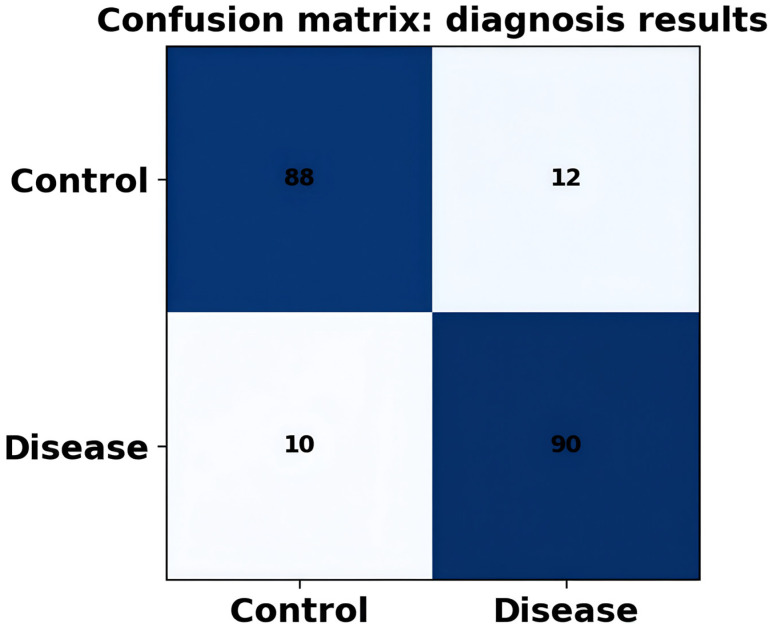
Confusion matrix showing classification performance distinguishing disease and control microbiome samples accurately.

Figure 9 represents the results of classification in the control and disease cohort. Large diagonal values show that the classifications are correct and low off-diagonal values denote low misclassification. The performance of DysbioFormer is consistent with high predictive accuracy, which proves the good capturing of disease signatures. It is worth mentioning that disease class prediction is counterbalanced with the control class recognition, which is a sign of the model generalizability. This type of confusion matrix is useful in the quantitative assessment of classification measures (accuracy, recall, precision). The visualization offers a sensible description of diagnostic performance, which is required to justify the use of models in clinical research.

### 4.7. Explainability and Taxonomic Biomarker Discovery

Explainability DysbioFormer is an effective method that enables the discovery of biomarkers of taxonomy by directly measuring the role of individual microbial taxa in disease predictions using attribution mechanisms relying on attention. In particular, the summation of Set Attention Block (SAB) weights on a layer-by-layer basis and head-by-head basis is used to identify the taxa that repeatedly appear to have a strong contextual impact across microbial communities of disease-associated microbiology. Simultaneously, Pooling-by-Multihead Attention (PMA) seed–taxon affinity scores measure the contribution of specific taxon to the disease signature embedding world-wide. The taxa with high and consistent scores in attention in cross-validation folds as well as high PMA affinity scores are preferred as candidate biomarkers. These taxa are characterized as microbial aspects that recurrently lead to diagnostic choices and are not scattered indicators or cohort-related ones. Patterns of disease-specific enrichment or depletion that are seen in these high-importance taxa also provide further evidence of their applicability as a biomarker of dysbiosis. It is also interesting to note that a number of high-importance taxa found by DysbioFormer such as Bacteroides, Faecalibacterium, and Prevotella has been found in previous microbiome studies to consistently be biomarkers in colorectal cancer and inflammatory bowel disease, which is evidence that the extracted signatures are biologically plausible and have clinical relevance. This attention-based ranking system allows the orderly finding of strong, interpretable microbial biomarkers which can be used to drive downstream hypothesis testing in clinical settings, to conduct studies focused on specific validation of such biomarkers and precision diagnostics. DysbioFormer is a predictive framework that explicitly connects predictive results to both evolutionally and functionally sensible microbial taxa to further biomarker discovery beyond black-box classification to clinically actionable microbial signatures. The results of the extended attribution stability analysis and other biomarker validation findings can be observed in Supplementary Figures S11 and S12.

### 4.8. Comparative Evaluation

The DysbioFormer model shows better diagnostic capability compared to conventional machine learning paradigms and traditional transformer models for microbiota analysis. Unlike other conventional methods, DysbioFormer takes into consideration the set structure information of microorganisms present in a patient with a focus on PMA aggregation. The model succeeds in providing better accuracy and F1-score due to attention-focused relational learning, which aids in identifying meaningful relationships with patient diseases. The model shows better generalization capability compared to other existing diagnostic models based on microbiota.

Table 5 compares a comparative study of DysbioFormer to commonly used machine learning and AutoML methods in the literature of microbiome. The comparison shows a steady performance increase in the proposed framework in various assessment dimensions, which implies enhanced strength, discrimination, and reliability in predicting diseases using heterogeneous sets of microbiomes.

**Figure 10:**
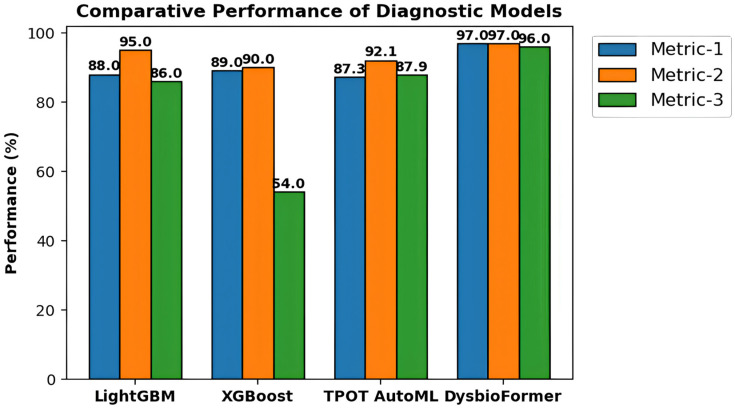
Comparative evaluation of diagnostic models demonstrating superior performance of DysbioFormer across cohorts.

Figure 10 illustrates how the diagnostic models performed in comparison to three assessment criteria. The results of DysbioFormer are always better compared to the traditional machine learning and AutoML methods, which indicates the capability to model multifaceted microbial interactions and produce reproducible and high-quality disease representations in heterogeneous cohorts.

### 4.9. Discussion

The general findings indicate that DysbioFormer is very discriminative in its ability to represent microbiome variation among heterogeneous cohorts; it is able to identify dysbiosis signatures at both taxon-specific and community-wide levels. The differences in taxonomic abundance, differences in diversity, and compositional gradients were always in line with previously known ecological patterns, which supported the biological plausibility of the learned embeddings. Diversity indices showed a distinct division between disease and control samples and CLR-transformed abundance distributions and variance stabilization indicated the consistency of the pre-processed data upon which downstream relational model analysis was performed. Indeed, quality-filtered and denoised sequence sets also helped to construct the robust token and high read-retention and significantly small error rates confirmed the integrity of the amplicon sequence variants. The harmonization using empirical Bayes reduced the cross-study effects and this was validated by better inter-cohort alignment in the PCA projections and lower dispersion in the study-specific abundance distributions. These results suggest that the harmonized datasets provide a stable basis for inference of transformer-driven multisets. Assessment of relational modeling by use of Set Attention Blocks showed significant interaction patterns between taxa and these interaction patterns captured ecologically consistent dependencies. PMA-aggregated signature vectors were clear in terms of disease and control embeddings as exhibited by high cohesion in pooled representation space. This encoded relationship resulted in high diagnostic accuracy, where DysbioFormer performed significantly higher in terms of accuracy, F1-score and AUC than previous microbiome-based standards. A key limitation of the study is that all of the assessments were performed in the harmonized MicrobiomeHD collection, and this lack of independent validation of entirely independent cohorts means that the results cannot be generalized to unobservable populations and sequencing platforms. The comparative findings are now clearly introduced as references to the context only and the restrictions to differences in datasets and assessment protocols are clearly noted. Although other models are able to capture microbial indicators that are of interest to varying extents, their performance is significantly lower than that of DysbioFormer that attained 97% accuracy, approximately 97% AUC, and approximately 96% F1-score. Compositional normalization, phylogenetic embeddings, multiset tokenization, and attention-driven relational inference are all useful to achieve the consistent diagnostic output across various cohorts. The obtained results corroborate that the architecture can predict disease-level information that is reliable and interpretable in microbial community architecture with high accuracy and predictive ability without prior information.

## 5. Conclusions and Future Work

This work aimed at solving the problem of existentially determining disease-related microbial patterns in heterogeneous data of human gut microbiomes, which is frequently complicated by compositional variability, batch effects, and complicated inter-taxon interactions. The DysbioFormer framework proposed was meant to learn these multilevel microbial relationships using an integrated pipeline that integrates stringent preprocessing, phylogenetic embedding, multiset token-creation and attention-based relational modeling. The model uses the high-dimensional structure of a community that is not based on engineered microbial markers or handcrafted features to translate the high-dimensional community structure into coherent diagnostic representations. Here, the findings show that DysbioFormer provides significant predictive performance improvements relative to the well-established microbiome diagnostic bases, which obtain high levels of accuracy, F1-score, and AUC in a broad range of disease cohorts. Its capacity to maintain an evolutionary coherence, stabilize compositional variation and bring multi-study data into a harmonious state makes it possible to generalize, despite significant biological and technical dissimilarity. The learned representations are interpretable and biologically plausible as the extracted signatures indicate significant taxonomic contribution based on the existing literature on dysbiosis. The study also takes a step toward the next level of microbiome-guided diagnostics by integrating compositional, phylogenetic, and relational evidence to the extent of increased reliability and mechanistic understanding. The study highlights the promise of transformer-based multiset modeling to aid in the future in terms of the discovery of microbial biomarkers and early disease detection. Altogether, the results confirm that DysbioFormer provides an effective computational basis of scalable, data-oriented characterization of microbial dysbiosis and can add meaningful methodological and practical contributions to microbiome informatics. This study is restricted to 16S rRNA sequencing data and has a significant amount of computation requirements. The next steps to be taken are validation on completely independent external cohorts and extrapolation to shotgun metagenomic data.

The directions of the future are towards longitudinal AI-based microbiome monitoring, which makes it possible to profile the changes in the microbiome over time to enhance the sensitivity of detection of early illnesses. Incorporation of multi-omics integration—integration of metatranscriptomics, metabolomics as well as host immunogenomic signals—into the framework will add more interpretability of the mechanisms and increase phenotype resolution. Trends such as the creation of AI-enabled point-of-care diagnostic hardware and the integration of optimized microbial signature classifiers into compact hardware to support clinical decisions in a few seconds are all part of the so-called translation paths. Precision-oriented expansion entails individualized microbiome-based treatment, in which adaptive models suggest individualized treatment modulation schemes. Lastly, to achieve strong external validation, it is essential to have multicenter clinical validation of the framework in cohorts spread across geographic regions in order to demonstrate the extent of generalizability, reduce site-specific biases, and raise the framework to regulatory-level diagnostic reliability fit to deploy in real-world healthcare settings.

## Figures and Tables

**Table 1:** Comparative summary of microbiome-based diagnostic models.

Study	Disease Scope	Data Source/Cohorts	Model Paradigm	Interpretability	Cross-Cohort Generalization	Compositionality/Batch Handling	Strengths	Limitations
Rahman et al. [17]	Five cancers	Cancer microbiome cohorts	ML + XGBoost	SHAP	Moderate	CLR + filtering	Explainable, strong tabular ML	Genus-level only, limited transfer
Song and Zhou [18]	CRC, Crohn’s, Immunotherapy	Multi-study cohorts	RF	Feature importance	Partial	Cross-study leveraging	Handles heterogeneity	Needs dataset overlap, batch-sensitive
Ram Das et al. [19]	Poultry pathogens	Farm microbiome + metadata	Transformer	Attention + PageRank	Low	Not addressed	Attention-based explanations	Domain-specific, metadata-dependent
Guo et al. [20]	CRC, IBD	HMDAD, Disbiome	GNN + LightGBM	Node importance	Low–Moderate	Not explicit	Models nonlinear relations	Graph completeness dependency
Kim et al. [21]	IBD	Multicenter cohorts	sPLS-DA	Feature weights	Low	CLR	High diagnostic power	Binary task, cohort-specific
Boodaghi–izaji et al. [22]	Multidisease	Stool microbiome	ML	Feature ranking	Low	Not explicit	Multidisease capability	Inter-individual variability
Syama et al. [23]	IBD, CRC	Metagenomic datasets	GraphSAGE + Boosting	None	Moderate	Not explicit	Graph-based modeling	Sensitive to sparsity
Wang et al. [24]	Multidisease	Human microbiome datasets	PM-CNN	None	Low–Moderate	Not explicit	Uses evolutionary structure	High computation
Proposed DysbioFormer	IBD, CRC, HIV, CDI, Obesity	MicrobiomeHD	Multiset Transformer	Attention weights + PMA	High	CLR + Empirical Bayes	Structure-aware, transferable, interpretable	Requires transformer training resources

**Table 2:** Representative MicrobiomeHD disease subsets.

Dataset ID	Condition	Case Count	Control Count
ibd_gevers	Crohn’s disease	224	31
crc_baxter	Colorectal cancer	120	172
hiv_noguerajulian	HIV	206	34
ob_goodrich	Obesity	183	433
cdi_schubert	Clostridioides difficile	94	155

**Table 3:** Cohort summary statistics.

Cohort	Sample Count	Avg. Age	Avg. BMI	Gender Ratio (M/F)
Control	120	35.4	23.1	60/60
Disease	130	36.1	25.4	62/68

**Table 4:** Simulation parameter and hardware setup.

Parameter	Value/Setting
Token Dimensionality	128
Number of SAB Layers	4
Attention Heads	8
Batch Size	64
Learning Rate	0.0005
Epochs	200
CLR Pseudo-count	1 × 10^−6^
Empirical Bayes λ	0.01
PMA Seed Vectors	16
Dropout	0.2

**Table 5:** Reported performance of representative microbiome diagnostic models from prior studies.

Model	Accuracy (%)	AUC/AUC-ROC	F1-Score (%)
Optimized LightGBM (MS) [26]	88	95	86
XGBoost Multiclass (AIDs) [27]	89 (mean)	90 (macro)	54 (mean)
TPOT AutoML (ME/CFS) [28]	87.3	92.1	87.9
Proposed DysbioFormer	97	97	96

## Data Availability

The datasets of microbiomes analyzed in this study are publicly available through the MicrobiomeHD [25] repository. The results obtained are processed and contained in the manuscript and additional files.

## Supplementary Materials

The following supporting information can be downloaded at https://www.mdpi.com/article/10.3390/diagnostics16050688/s1.

## Abbreviations

SAB	Set Attention Block
PMA	Pooling by Multihead Attention
SHAP	SHapley Additive exPlanations
ESCA	Esophageal Carcinoma
COAD	Colon Adenocarcinoma
DeepLIFT	Deep Learning Important FeaTures
HG-LGBM	Heterogeneous Graph-Light Gradient Boosting Machine
LightGBM	Light Gradient Boosting Machine
HMDAD	Human Microbiome Disease Association Database
OTU	Operational Taxonomic Unit
