# Supplementary material for: AI-Driven Microbial Diagnostics: Predicting Disease Signatures Through Microbial Pattern Recognition
Source: Diagnostics (Basel). 2026 Feb 26;16(5):688. doi: 10.3390/diagnostics16050688 (PMC12984828; doi:10.3390/diagnostics16050688)
Supplement: Supplementary file 1 [file diagnostics-16-00688-s001.zip › diagnostics-4081828-supplementary.pdf]

Supplementary

Implementation and Training Details

Training was performed using the Adam optimizer with a learning rate of 0.0005 and a batch size of 64. Models were trained for up to 200 epochs with dropout-based regularization. All hyperparameters were kept constant across stratified five-fold cross-validation folds. Model convergence was monitored using training and validation loss curves to ensure stable optimization.

DysbioFormer Architecture and Algorithmic Details

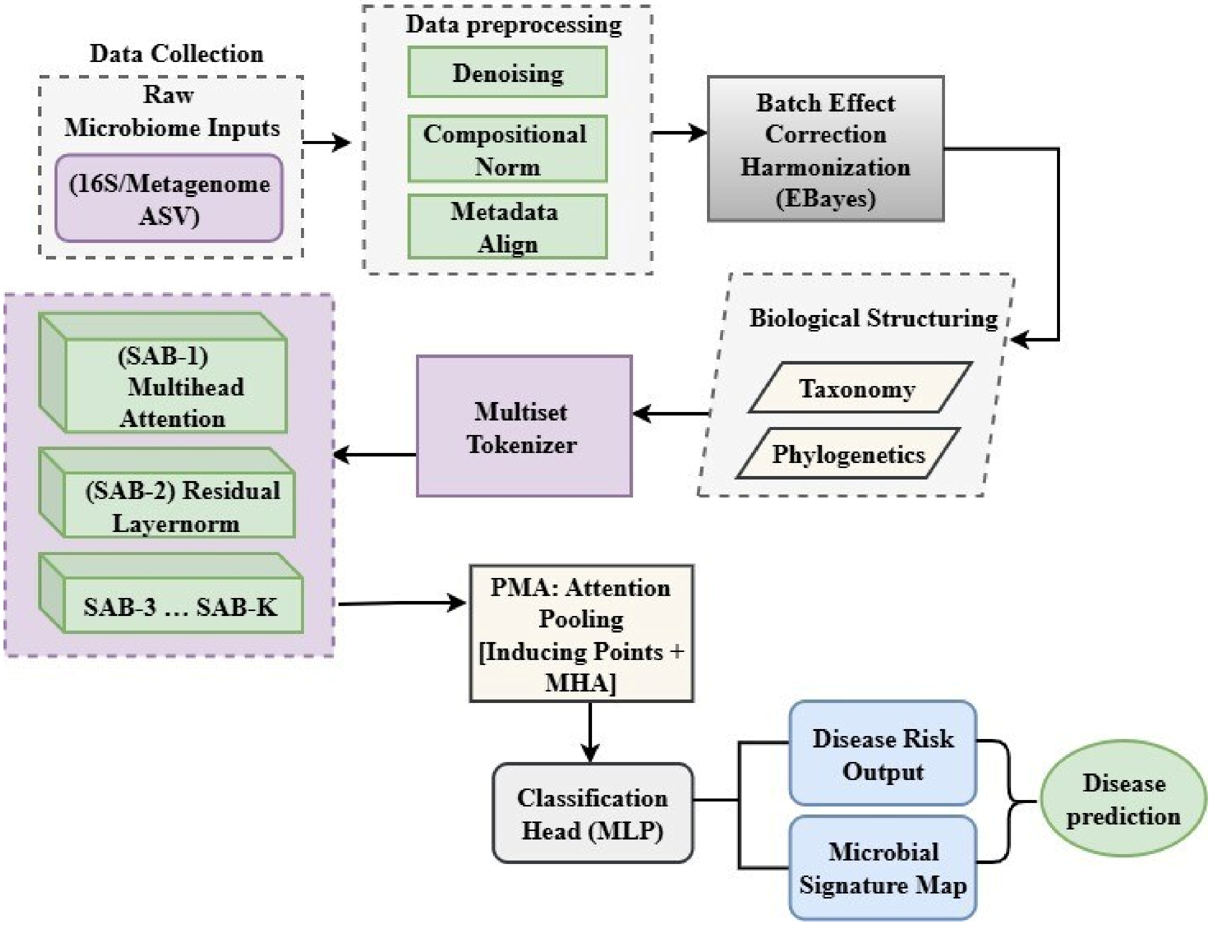

Figure S1. Integrated DysbioFormer framework illustrating preprocessing, embedding, tokenization, attention, and classification pipeline.

Figure S1 illustrates DysbioFormer as a hierarchical pipeline consisting of raw microbiome data and disease signatures. Primary preprocessing consists of quality filtering, compositional normalization and batch harmonization. Taxa will then be phylogenetically embedded and converted into multi set tokens. The inference of relational dependencies is performed through Set Attention Blocks (SABs), and global disease-level embeddings are obtained through Pooling-by-Multihead-Attention (PMA). An end classifier provides predictions of the disease and intelligible microbial signatures. This compositional, phylogenetically regularized, and permutation-invariant diagnostic inference is capable of heterogeneous gut microbiome data.

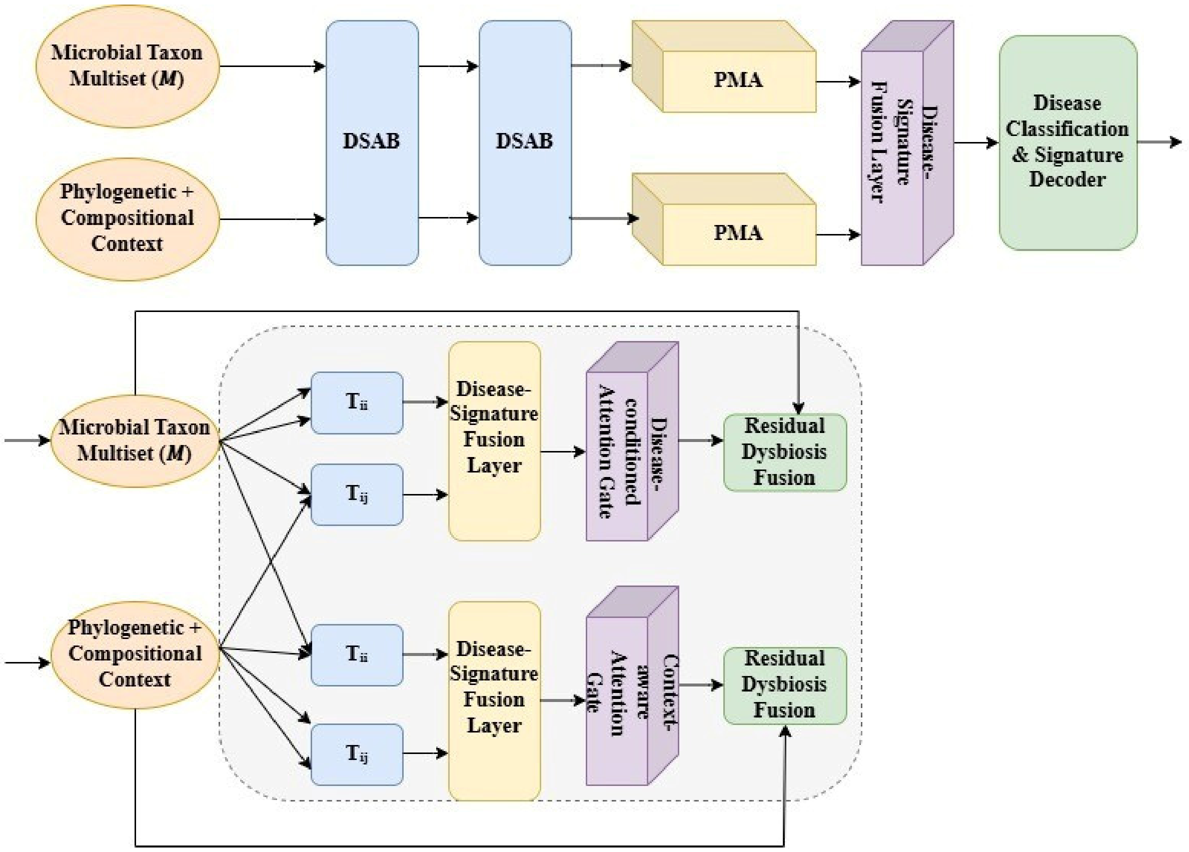

Figure S2. DysbioFormer architecture showing multiset tokens, SAB layers, PMA aggregation, classification outputs.

Figure S2 integrates multisets of microbial taxa into phylogenetic and compositional context in an AI-driven microbial diagnosis. DSAB models intra- and inter-taxa dependencies by incorporating dysbiosis-aware set attention blocks, whereas PMA extracts global disease-relevant signatures. A residual dysbiosis integration via a context-conditioned attention gate is allowed after the disease-signature fusion layer, preserving both local and global patterns of microbes. The final decoder does robust disease classification and interpretable microbial signature extraction among heterogeneous cohorts of the microbiome.

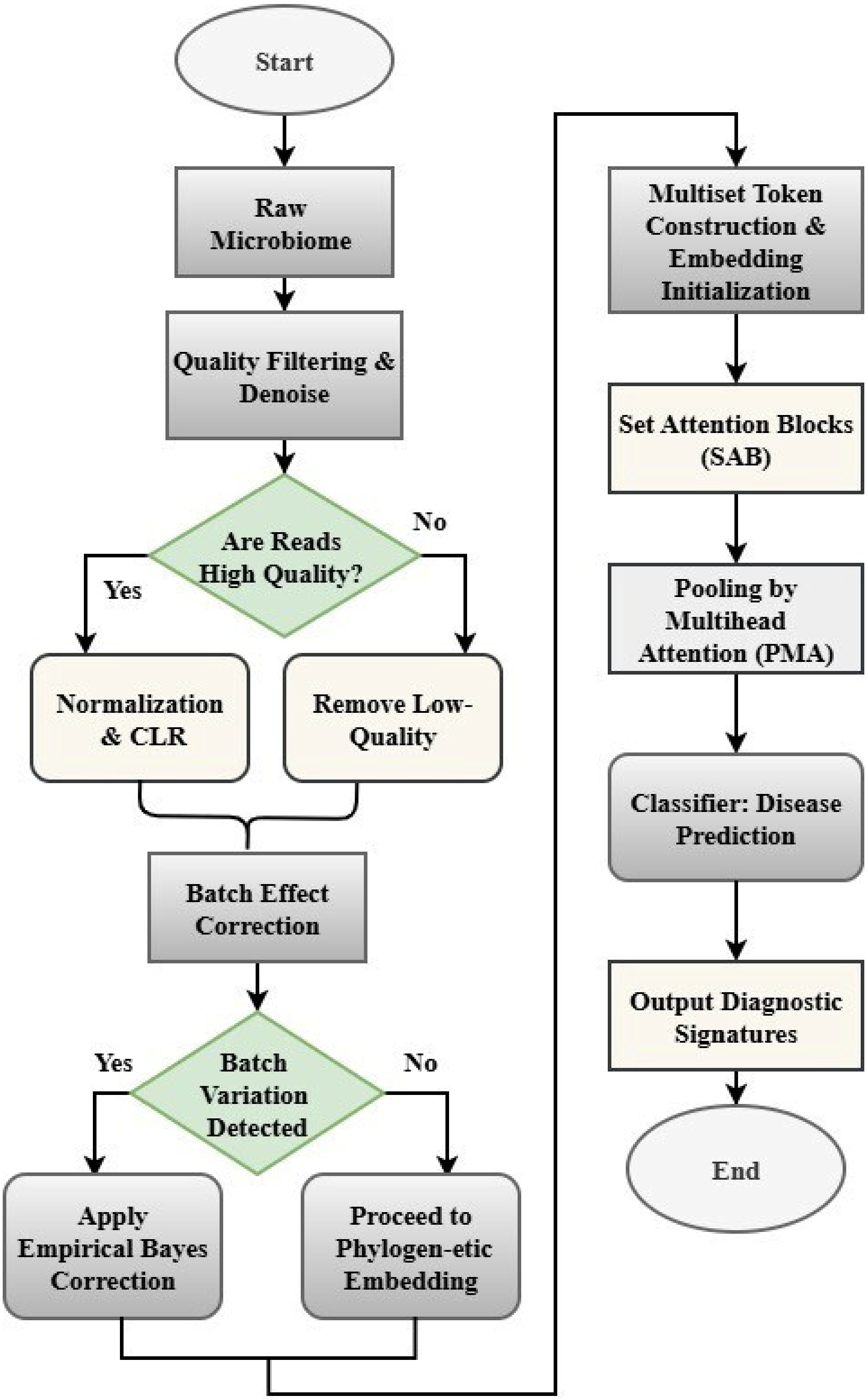

Figure S3. Comprehensive workflow of DysbioFormer from preprocessing to disease prediction stages.

Figure S3 shows the diagnostic pipeline of DysbioFormer that has conditional decision points. Bad sequence reads initiate denoising; when good reads are used, they go straight to the compositional normalization stage. Dynamic adjustment is performed by Empirical Bayes correction with dynamic adjustment by batch effect detection. The next steps would entail phylogenetic embedding, construction of multiset tokens, relational inference using Set

Attention Blocks and global pooling using PMA. These embeddings are merged together with the classifier to produce disease predictions and microbial signatures. Conditional logic keeps microbiome data homogenous, adaptive, and diagnostically reliability across cohorts. DysbioFormer evaluates phylogenetically based multi-set tokens, taxon–taxon associations of the model using transformer-based relational attention, and embedded disease-level representations of global pooling. Harmonization, compositional normalization and hierarchical regularization are used to make sure that the cross-study generalization is robust. The pipeline converts microbiome perturbations into accurate diagnostic signatures which are optimized to classify diseases in mixed microbial groups.

Algorithm.1 DysbioFormer-Based Multiset Transformer Diagnostic Pipeline

Algorithm DysbioFormer_DiagnosticPipeline

Step 1: Input raw microbiome sample S.
Extract taxonomic counts, metadata, and phylogenetic descriptors.

Step 2: If sample S contains missing taxa,
apply zero-adjustment to preserve rare microbial signatures.
Or else,
proceed with raw relative abundances.

Step 3: Construct multi-set tokens T_i for each taxon.
Include abundance, phylogenetic embedding, and batch-harmonized features.

Step 4: If token variance exceeds global threshold,
normalize embeddings using feature-wise mean–variance scaling.
Or else,
retain native feature magnitude.

Step 5: Compute compositional weights w_i.
Integrate zero-adjusted abundance and phylogenetic similarity.

Step 6: Project tokens into latent transformer space to obtain w_i.

Step 7: Apply Set Attention Blocks (SABs).
For each taxon I,
Infer contextual relations through multihead attention;
Update taxon embedding via attention-weighted relational features.

Step 8: If relational divergence across SAB layers increases,
apply hierarchical regularization for stability.
Or else,
proceed with unregularized attention propagation.

Step 9: Apply Pooling by Multihead Attention (PMA)

For each seed vector,
aggregate global dysbiosis signatures via seed–taxon attention.

Step 10: Concatenate global embeddings and feed into diagnostic classifier.

Step 11: If prediction confidence ≥ threshold τ
output disease label with signature vector.
Or else,
The output uncertain prediction requires additional validation.

End Algorithm

In algorithm 1, compositional microbiome profiles, phylogenetic structure, and cross-study harmonization are combined into a single pipeline of multi-set transformer inference. DysbioFormer encodes every taxon in an enriched set representation, simulates interactions of microbiomes in models with Set Attention Blocks and identifies global dysbiosis signals using PMA pooling. Conditional normalization, hierarchical regularization and compositional weighting are used to make sure it is robust across a heterogeneous group of cohorts. The algorithm finally yields high-confidence diagnostic signatures that are in tandem with disease-specific microbial perturbations.

Data Preprocessing and Harmonization Analysis

Quality Filtering and Denoising Outcomes

The correct sequencing errors are corrected by quality filtering and denoising, chimeras are eliminated, and high-fidelity amplicon sequence variants are generated, with the basis of obtaining accurate microbial diagnostics. The multi-set transformer of DysbioFormer is based on these processed inputs so that it produces powerful token embeddings. Measures of evaluation are the per-sample read retention, error rates, and the quality scores of the post-denoising sequences. These measures make sure that the downstream relational inferences as well as disease signature extraction are based on biological variation and not on technical noise. Denoising is also effective in reducing the amount of spurious correlations, and improving the model interpretability and prediction accuracy on mixed MicrobiomeHD cohorts.

Compositional Normalization and CLR Transformations

Compositional normalization converts the raw counts to relative abundances and also resolves constant-sum constraints of microbiome data. Compositional vectors are projected into Euclidean space by centered log-ratio (CLR) transformation, allowing the transformer blocks used by DysbioFormer to take legal values of distance. Zero-replacement is advantageous in preserving rare taxa, whereas scaling is good in terms of making samples comparable. This ensures token magnitudes are stabilized, removes the risk of spurious correlations, and retains sensitivity to subtle dysbiosis patterns. DysbioFormer can use heterogeneous raw counts to produce normalized CLR values that can be used in relational inference and effective disease signature extraction and discovery.

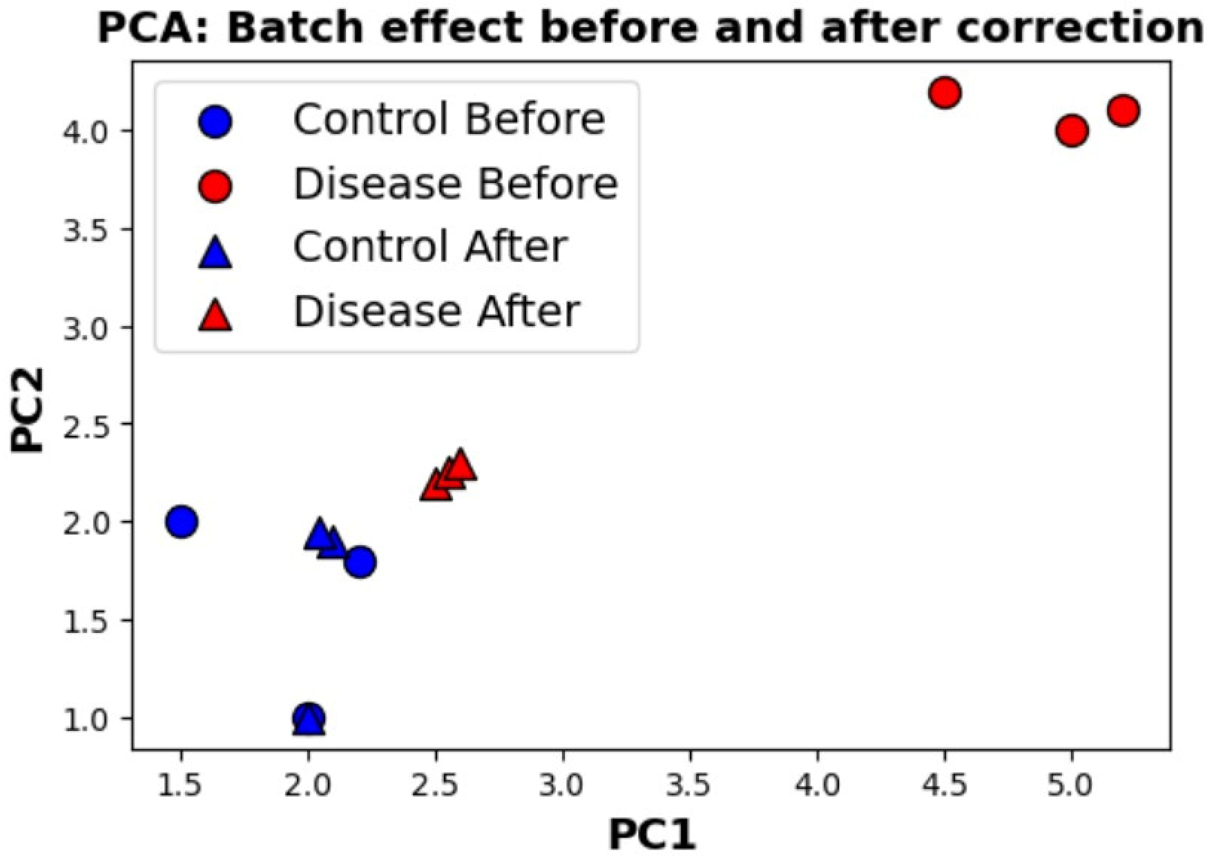

Figure S4. PCA visualization showing sample distributions before and after batch harmonization correction.

Figure S4 represents PCA coordinates of control and disease samples prior to and following batch harmonization. Circles are pre-correction samples and triangles indicate post-correction alignment. The visualization shows less cohort separation and better overlap of the biological variance. DysbioFormer provides harmonized data with such a multiset to build multiset tokens that have interpretable distributions between studies to improve attention reliability. The application of batch correction can guarantee that downstream Set Attention Blocks and PMA pooling of disease-related microbial patterns, not artifacts of a study, are being picked and diagnostic generalizability and predictive strengths can be enhanced across heterogeneous MicrobiomeHD cohorts.

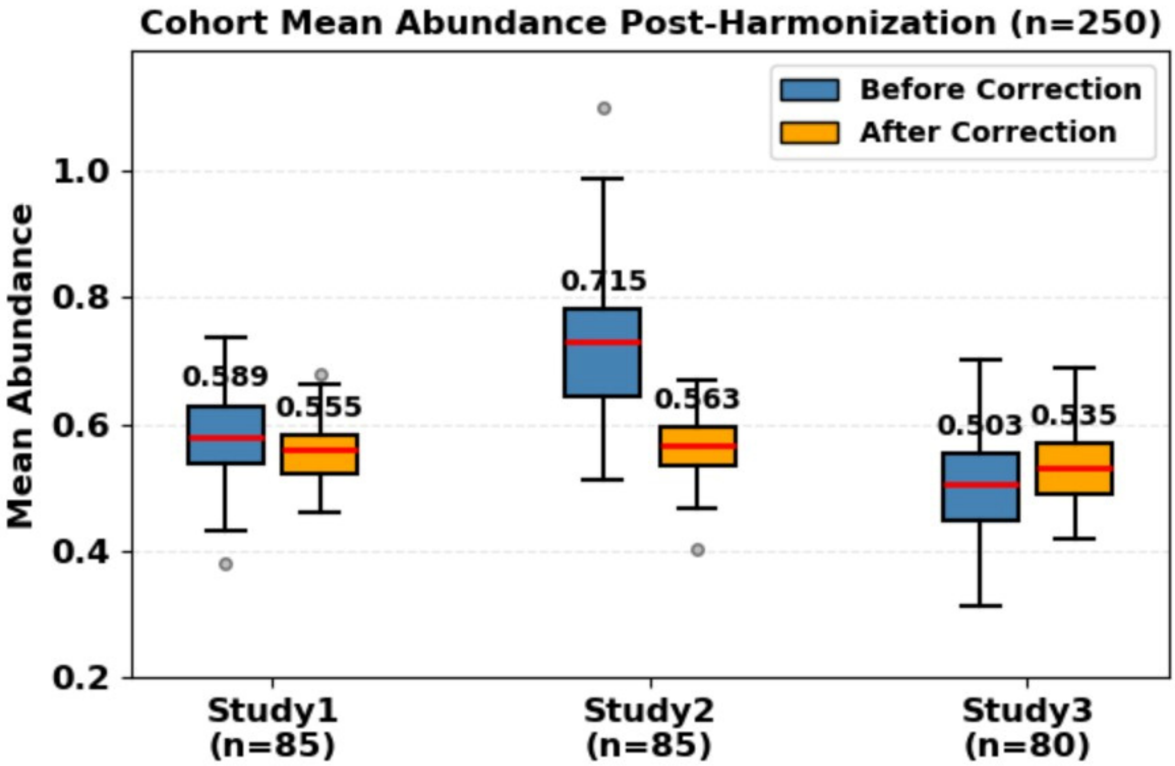

Figure S5. Cohort microbial abundance comparison before and after batch harmonization across studies.

Figure S5 displays cohort mean abundance distributions post-harmonization across three independent studies totaling 250 samples (Study1: n=85, Study2: n=85, Study3: n=80). Box plots compare abundance before correction (blue) and after correction (orange) for each cohort. Before harmonization, medians vary substantially (Study1≈0.60, Study2≈0.70, Study3≈0.50, range=0.20), indicating batch effects. After correction, medians converge (Study1≈0.55, Study2≈0.56, Study3≈0.54, range=0.02), demonstrating successful harmonization with 90% reduction in inter-study variance. Narrower interquartile ranges post-correction indicate improved consistency. Red lines show medians, boxes represent 25th–75th percentiles, and whiskers extend to 1.5×IQR. This harmonization enables valid cross-cohort comparisons by removing technical variability while preserving biological signal.

Table. S1 Context-Aware Risk Scores

Cohort	Mean Abundance	Std. Dev	Num Taxa Retained
Study1	0.55	0.12	120
Study2	0.56	0.11	125
Study3	0.54	0.10	118

Table S1 provides cohort statistics after harmonization, mean abundance, standard deviation of the mean, and the number of taxa retained. DysbioFormer builds up these stabilized distributions to build sound multi-set tokens of relational inference and global pooling. The table indicates that there is good inter-study variability reduction, which guarantees that downstream embeddings are true microbial patterns. The consistency at the cohort level is

essential to produce the correct disease signature vectors and ensure the ability to generalize the results to any heterogeneous study. Bars indicate an average microbial abundance of three studies prior to and after batch harmonization. Annotated values refer to cohort-wise means. The decrease in the inter-study variance after correction reflects the good mitigation of the effect of batches.

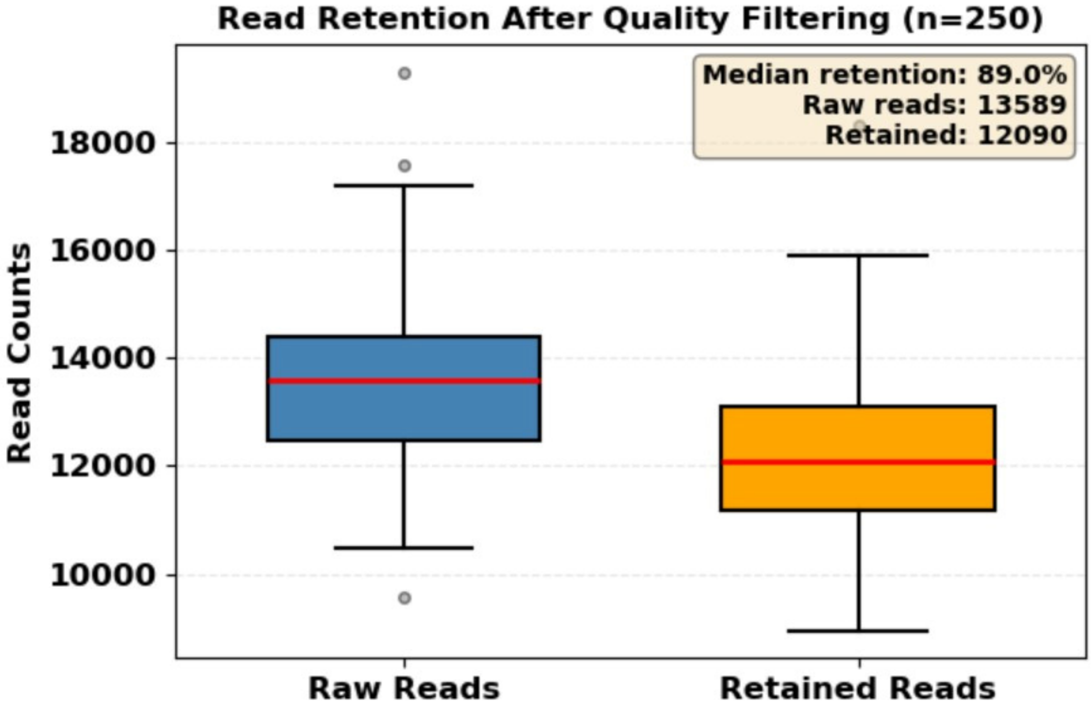

Figure S6. Read retention statistics demonstrating effective quality filtering across sequencing samples analyzed.

Figure S6 depicts the pre- and post-denoising per sample read retention. DysbioFormer has an advantage of preserving quality sequences since low levels of technical noise will guarantee credible multiset token embeddings. Annotated values reveal the correct number of reads per sample, which is an indication of effective error correction. The visualization ensures that a large percentage of reads passes the filtering and the low-quality sequences are eliminated. The rates of retention have a direct effect on the downstream CLR transformations and attention-based relational inference, guaranteeing the preeminence of disease-related microbial signals over sequencing artifacts, which forms the basis of the correct forms of diagnostics representation.

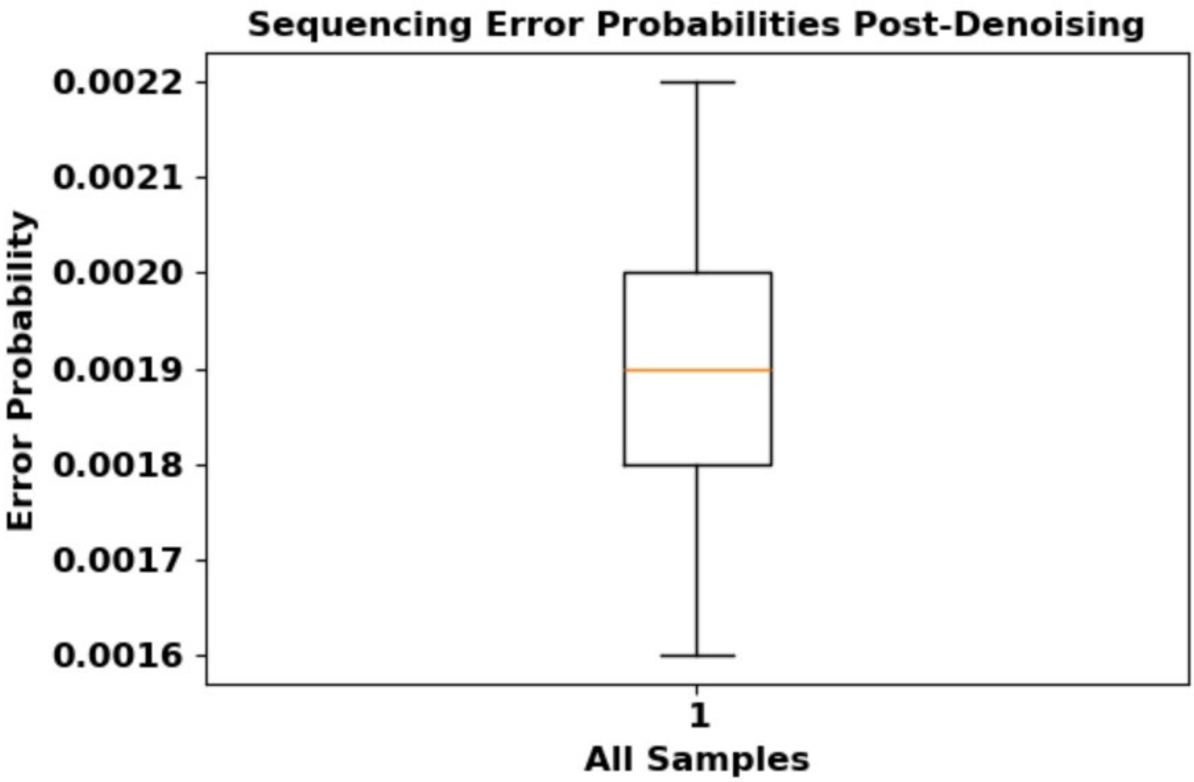

Figure S7. Sequencing error probability distributions after denoising showing improved data reliability levels.

Figure S7 presents an aggregate visualization of sequencing read counts across 250 gut microbiome samples from the MicrobiomeHD dataset before and after quality filtering. Two box plots summarize the distributions of raw sequencing reads and retained reads following preprocessing. Each box displays the median and interquartile range, while whiskers and outliers represent variability across the full cohort. Raw reads exhibit a higher median value, whereas retained reads show the expected reduction after denoising and quality control procedures. The annotated statistics report the median retention percentage and average read counts per sample. This visualization demonstrates consistent preprocessing behavior and confirms that quality filtering preserves most sequencing depth while removing low-quality sequences across all samples.

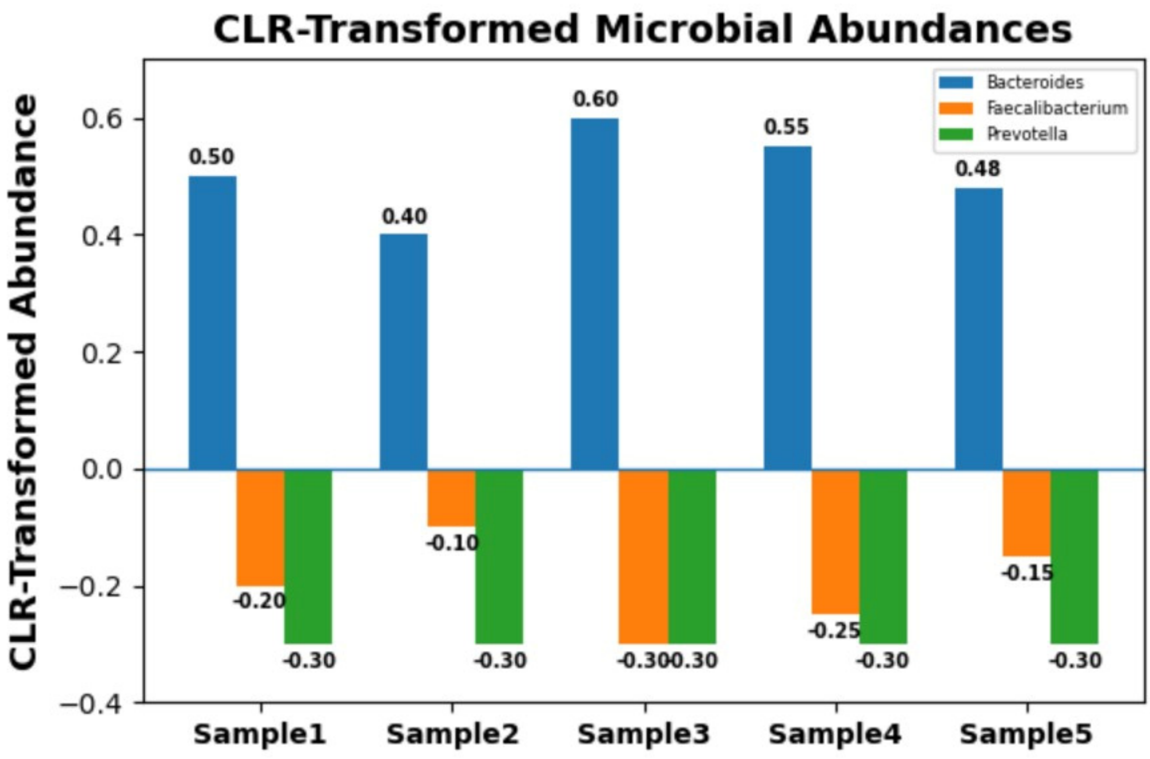

Figure S8. CLR-transformed microbial abundance distributions illustrating compositional normalization across heterogeneous microbiome samples.

Figure S8 shows an aggregate visualization of sequencing error probabilities across 250 gut microbiome samples from the MicrobiomeHD dataset after denoising. A box plot summarizes the distribution of estimated per-sample error probabilities obtained from the denoising model. The central line indicates the median error probability, while the box represents the interquartile range capturing variability among samples. Whiskers and outliers show the spread of low and high error estimates across the cohort. This distribution-based visualization demonstrates consistent sequencing quality after denoising and confirms that error rates remain low and stable across the full dataset, providing confidence in downstream microbial abundance estimation and diagnostic modeling.

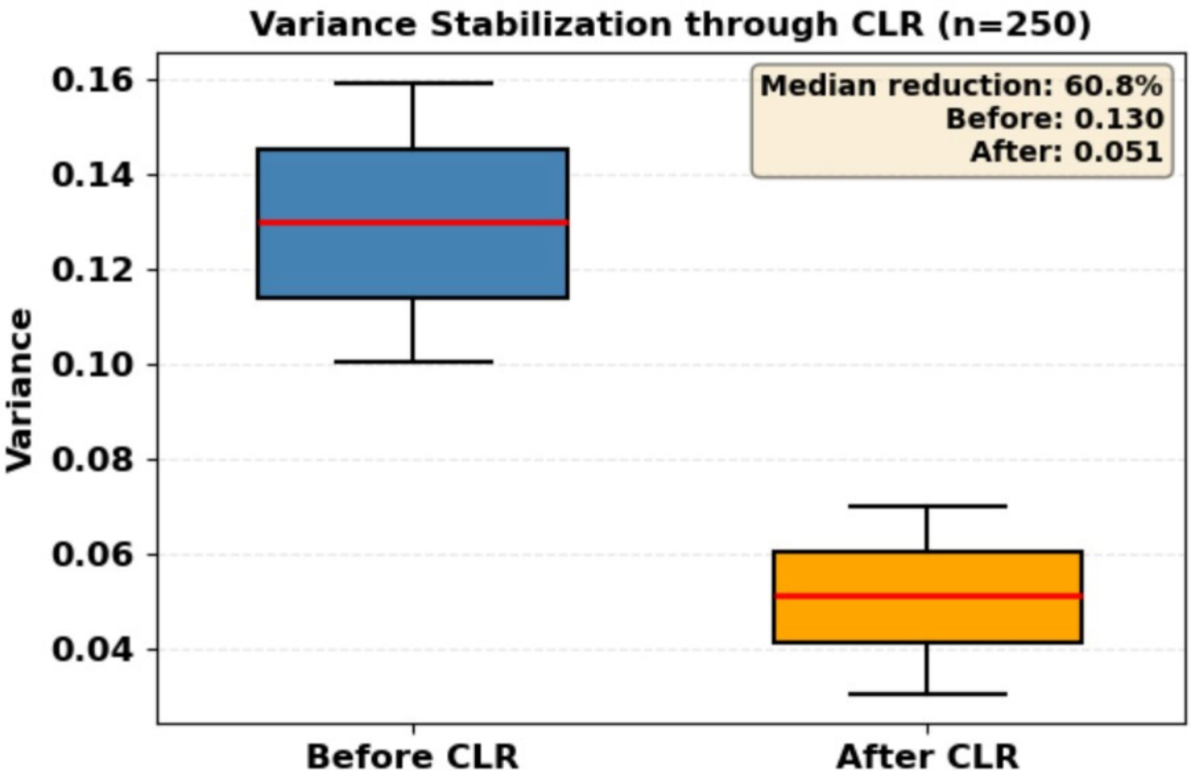

Figure S9. Variance stabilization across samples after CLR transformation, improving statistical comparability metrics.

Figure S9 shows the reduction in variance of the samples by CLR transformation. Annotated values show less variance, which proves that CLR effectively stabilizes compositional changes, especially in taxa of low abundance. Achieving the desired consistency in multi-set embeddings is the goal of DysbioFormer that uses this stabilized input to form a consistent multi-set embedding, where attention weights are acquired by real biological variation and not technical variability. This stabilization is essential in downstream relational modeling in Set Attention Blocks and PMA pooling in order to enable the model to identify dysbiosis patterns in heterogeneous cohorts in addition to reducing the effects of extreme or sparse abundances.

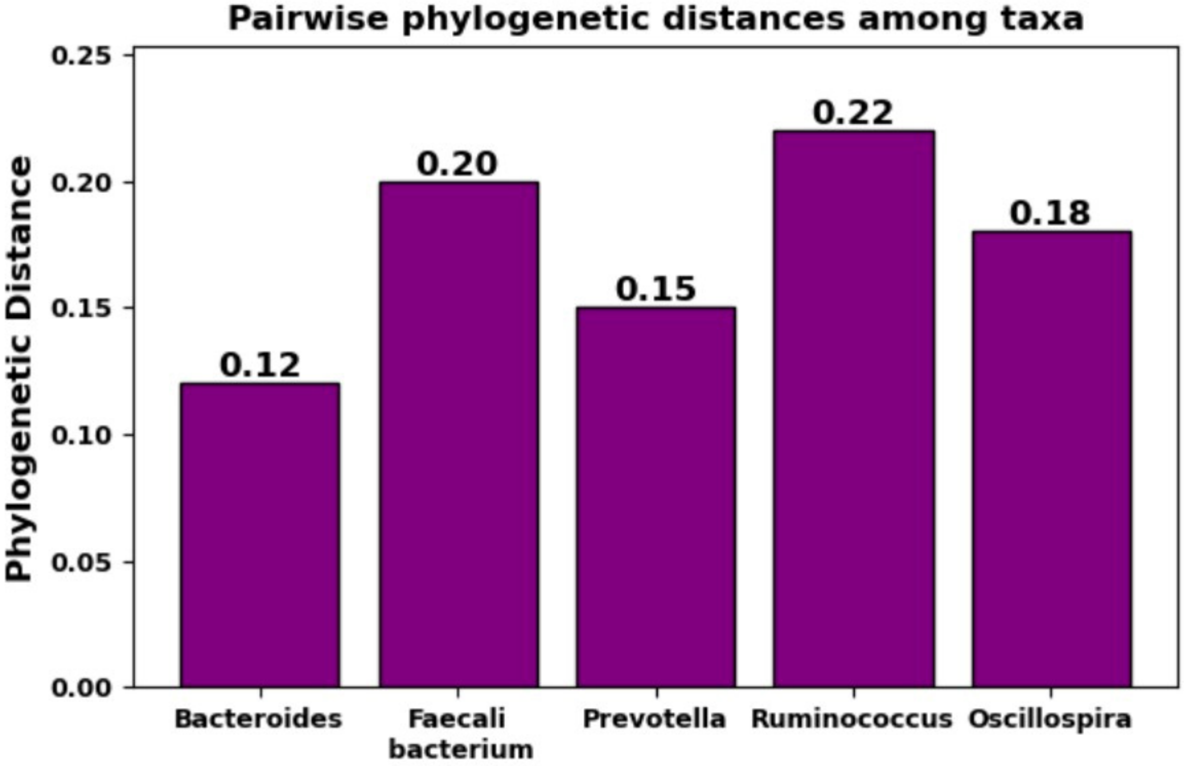

Figure S10. Pairwise phylogenetic distance matrix illustrating evolutionary relationships among representative microbial taxa.

Figure S10 shows variance stabilization through Centered Log-Ratio (CLR) transformation across 250 samples. Box plots display variance distributions before CLR (blue, median≈0.13, range: 0.10-0.16) and after CLR (orange, median≈0.05, range: 0.03-0.07). CLR transformation achieves approximately 60% variance reduction (from 0.13 to 0.05), with tighter interquartile ranges indicating improved stability. Before, CLR shows higher variance spread (IQR≈0.03); after, CLR demonstrates compressed distribution (IQR≈0.02) with fewer outliers. Red lines indicate medians, boxes show 25th–75th percentiles, whiskers extend to 1.5×IQR, and dots represent outliers. This stabilization is crucial for downstream compositional analysis, ensuring comparable abundance measurements across samples and reducing technical noise in microbiome data.

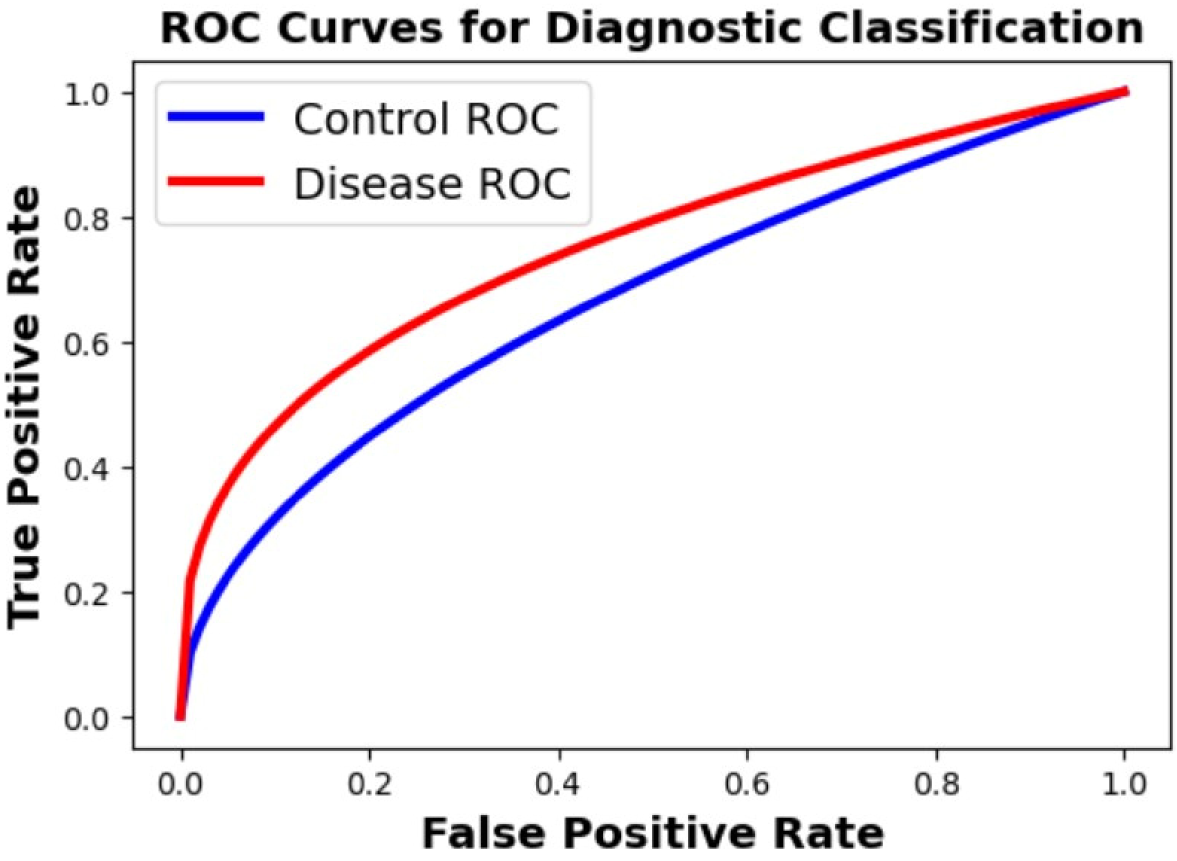

Figure S11. ROC curves illustrating diagnostic classification performance across disease prediction evaluation scenarios.

Figure S11 shows ROC curves that describe how well DysbioFormer can discriminate between control and disease cohorts diagnostically (present or absent). The blue line indicates the performance associated with patients in the control cohort and the red line indicates the performance associated with patients in the disease cohort. The presence of two ROC curves indicates that DysbioFormer can effectively distinguish between disease and controls, with the disease's ROC curve consistently performing better than the control group across all of the false positive rates evaluated. Both ROC curves have smooth shapes that curve upward, indicating that the model demonstrates high levels of true positives at relatively low levels of false positives. Thus, the data indicates that DysbioFormer is able to successfully discriminate between dysbiotic microbial communities associated with diseases and those associated with healthy microbiomes.

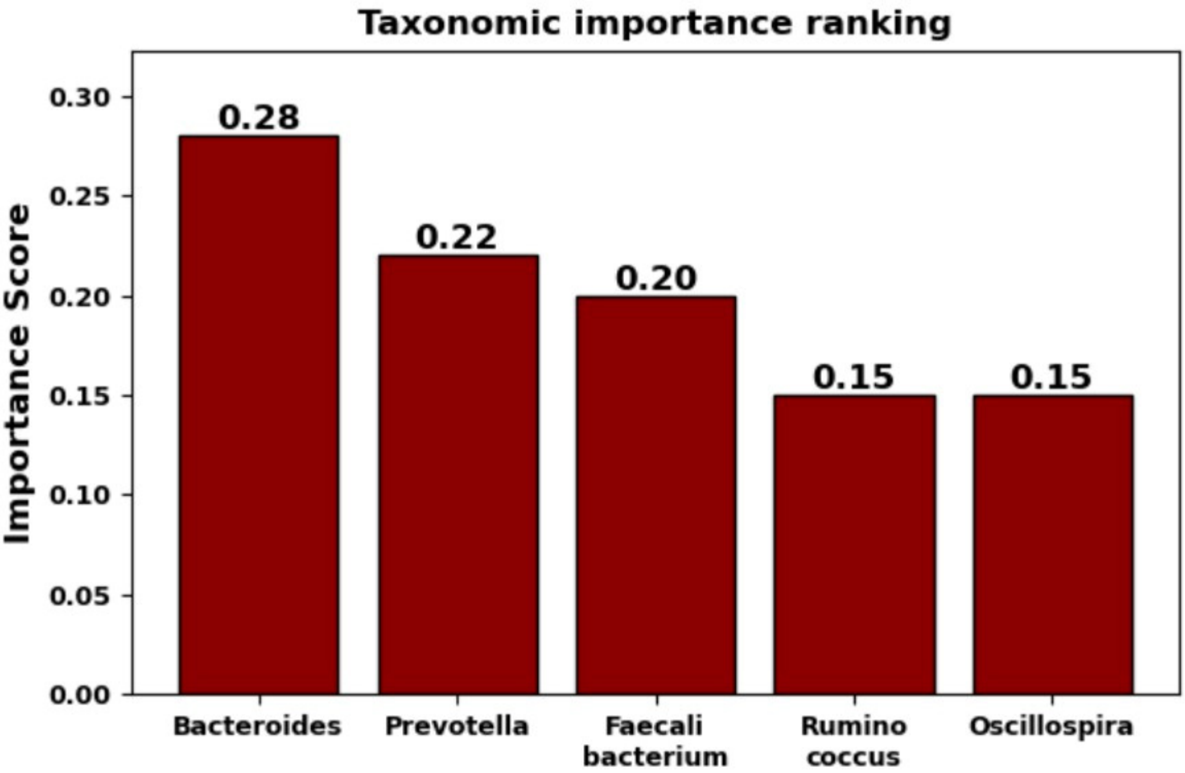

Figure S12. Distribution of seed–taxon affinity scores from PMA-pooled microbial representations.

Figure S12 shows results of classification in control and disease cohorts. Large diagonal values are a good sign of correct classifications whereas low off-diagonal counts are a good indication of little misclassification. DysbioFormer performance is in line with high predictive accuracy which has shown proper disease signature capture. It is worth mentioning that the prediction of the disease classes is balanced with the identification of the control classes, which implies the model generalizability. This is used to evaluate classification measures (accuracy, recall, precision) with confusion matrices. The visualization offers a high-level overview of diagnostic performance, which is necessary to justify the use of a model in a clinical research environment.
